# Patient safety and predictors for subsequent healthcare contact after self-care referral from Swedish ambulance services: a retrospective cohort study

**DOI:** 10.1186/s12873-026-01561-4

**Published:** 2026-04-02

**Authors:** Cecilia Fager, Håkan Johansson, Andreas Rantala, Anders Svensson, Mats Holmberg, Kristoffer Wibring, Anders Bremer

**Affiliations:** 1 Department of Health and Caring Sciences, Faculty of Health and Life Sciences, Linnaeus University, Kalmar, Växjö, Sweden; 2 Centre of Interprofessional Collaboration within Emergency Care (CICE), Linnaeus University, Växjö, Sweden; 3 Department of Ambulance Service, Region Kalmar County, Kalmar, Sweden; 4 Department of Research, Region Kalmar County, Kalmar, Sweden; 5 Department of Health Sciences, Faculty of Medicine, Lund University, Lund, Sweden; 6 Ambulance Service Department, Office of Medical Services, Region Skåne, Helsingborg, Sweden; 7 Department of Ambulance Service, Region Sörmland, Katrineholm, Sweden; 8 Center for Clinical Research Sörmland, Uppsala University, Mälarsjukhuset, Eskilstuna, Sweden; 9 Institute of Health and Care Sciences, Sahlgrenska Academy, University of Gothenburg, Gothenburg, Sweden; 10 Department of Ambulance and Prehospital Care, Region Halland, Halmstad, Sweden

**Keywords:** Ambulance service, Non-conveyance, Patient safety, Subsequent healthcare contact, Self-care referral, Mortality, Decision support systems

## Abstract

**Background:**

Ambulance clinicians increasingly refer patients to self-care, positioning ambulance service within a wider gatekeeping role and necessitating the assessment of whether self-care is a safe option or if further support is needed. Contrary to primary healthcare centre (PHC) referrals, self-care referrals often lack structured follow-ups, with the decision consequences remaining underexplored. This study investigates the outcomes and predictors of subsequent healthcare contact (SHC) and mortality among patients referred to self-care by ambulance services in three Swedish regions.

**Methods:**

This retrospective cohort study included 6,452 (954 children and 5,498 adults) ambulance assignments between 1 January 2023 and 31 December 31 2023 that resulted in self-care referrals. The primary outcome was SHC (PHC visits, ambulance service recontact, emergency department visits, and hospitalisations) and all-cause mortality within 72 h and 30 days. Bayesian multilevel logistic regression models were used to estimate the probability of recontact and mortality.

**Results:**

Approximately 30% and 25% of the adults and children sought SHC, respectively, with the majority being PHC visits. Mortality was < 2% within 30 days, and no deaths were observed among the children. Respiratory, infectious, and medical symptoms predominated among the children, compared with medical, surgical, and neurological symptoms among adults. Predictors of SHC included increased age, longer on-scene time, advisory decision support system (ADSS) use, and distance to hospital.

**Conclusion:**

Approximately one-third (30%) of patients sought SHC within 72 h of a self-care referral decision. Whilst this does not necessarily indicate adverse events, the lack follow-up and patient-reported data limits interpretation. Although the ADSS have the potential to reduce ED conveyance, they cannot replace nuanced clinical judgment, risking inequitable care for patients in rural areas, those with low health literacy, and frail older adults. Non‑conveyance decisions for patients aged 80 years or older should therefore be approached with heightened caution. Future research should explore patient perspectives to better understand re-engagement and improve the safety of self-care referrals.

**Supplementary Information:**

The online version contains supplementary material available at 10.1186/s12873-026-01561-4.

## Background

Patient perspectives of ambulance services are myriad: from merely a transport means to the nearest emergency department (ED) to recognising ambulance clinicians’ patient assessability, treatability, and safe on-scene dischargeability skills [1, 2]. Most patients who contact ambulance services anticipate ED conveyance, typically because their situation has become intolerable and they feel they have exhausted all other alternatives. However, when this perceived urgency is met by ambulance clinicians who view the call as ‘unnecessary’, patients have described problematic encounters characterised by degrading comments and a profound loss of dignity [3]. This expectational discrepancy may explain the varied patient experiences of on-scene discharge, including reassurance and confidence [4] or abandonment and rejection [1, 5–8].

The proportion of patients being discharged on-scene and not transported by the ambulance service after the on-scene assessment, a phenomenon known as ‘non-conveyance’, is increasing [4, 9, 10]. Evidence from a systematic review encompassing 67 studies within the ambulance service indicates that this is an international phenomenon spanning a wide range of healthcare systems. Despite variations in how ambulance services are organised across countries, non‑conveyance practices are shaped by common global pressures in emergency care, such as the lack of standardised decision‑making frameworks and the complexity inherent in assessing clinical risk [11]. This trend places greater demands on ambulance clinicians’ assessment skills, especially when no further physician-level expertise evaluation follows their initial assessment. Furthermore, non-conveyance decision-making requires knowledge of the appropriate level of care for each patient, such as a primary healthcare centre (PHC) referral or self-care advice [12–14].

The proportion of non-conveyance in the general patient population varies significantly between countries and regions (3.7–93.7%). Vulnerable patients, such as older adults, are more likely to be a part of the non-conveyed population [11]. Crucially, non-conveyance decisions do harbour patient safety risks. These risks have been explored by examining subsequent healthcare contacts (SHC), hospitalisation, and mortality across varying time-frames [10, 12, 15–18].

Within 48 h after a non-conveyance decision, 2.5–6.1% of patients recontact ambulance services, while 4.2–19.0% visit the ED [11, 16, 19]. A Danish retrospective registry-based cohort study examined outcomes for non-conveyed patients. Based on a sample size of 17,402 patients, approximately 5% of the patients were subsequently admitted to hospital within 48 h [16]. The mortality rate within 72 h after a non-conveyance decision is 0.08–6.1% [10, 11, 16, 19]. Longer distances to the nearest ED have been linked to a higher likelihood of non-conveyance [10]. Regarding the distribution of non-conveyance across the time of day, an increased frequency has been observed both during daytime and out-of-office hours [12, 14–18].

Self-care referrals are a common rationale for non-conveyance and well-recognised within healthcare frameworks as an appropriate level of care. A self-care referral decision requires ambulance clinicians to assess the patients’ ability to safely manage their own care and perform necessary activities to maintain or promote optimal health for acute or chronic conditions [20]. These assessments and decisions often present specific challenges. Research, including studies examining subsequent healthcare contacts, indicates that the complexity of non-conveyance decisions and their associated patient safety risks are shared across healthcare systems in Sweden and internationally [11, 21]. However, previous research on non-conveyance has primarily considered the patient population as a homogeneous group regardless of the level of care [11, 21, 22]. Although some sub-group analyses included children [23, 24], patients with trauma [25], and older adults [26, 27], a knowledge gap exists regarding patients referred to self-care. This is significant considering the frequent absence of structured follow-up after self-care referrals, in contrast to referrals to other levels of care, such as PHC.

Therefore, this study aimed to determine predictors of SHC and mortality among patients referred to self-care by ambulance services in three Swedish regions by exploring (a) the characteristics of patients; (b) the proportion of SHC or mortality within 72 h and mortality within 30 days; and (c) the predicted probability of SHC or mortality within 72 h and mortality within 30 days.

## Methods

### Study design and ethical approval

This retrospective cohort study was approved by the Swedish Ethical Review Authority (No. 2022-05223-01). Patient consent was not obtained because the researchers only had access to anonymized data. The study adheres to the Strengthening the Reporting of Observational Studies in Epidemiology (STROBE) guidelines [28] and the ROBUST (Reporting Of Bayes Used in Clinical Studies) framework [29, 30].

### Setting and context

This study was conducted in three Southern Sweden regions, consecutively including all patients referred to self-care by ambulance services over a 1-year period.

Ambulance services can be contacted via the national emergency number, 112. Ambulance assignments are prioritised by an Emergency Medical Communication Centre (EMCC) dispatcher: Priority 1 (acute, life-threatening), Priority 2 (acute, albeit not life-threatening), and Priority 3 (other assignments where reasonable waiting time is not expected to affect the patient’s condition) [31].

Ambulances are staffed with at least one registered nurse [32], and the national ratio of nurses to paramedics/emergency medical technicians is 80:20 [33]. These professionals are collectively referred to as ambulance clinicians in the present study. Ambulance teams apply non-conveyance guidelines, enabling ambulance clinicians to refer patients to alternative levels of care in addition to ED conveyance. The Rapid Emergency Triage and Treatment System (RETTS©) is the primary triage tool used in Sweden. It helps assess the patient’s clinical status via the early signs and symptoms (ESS) code (chief complaint) and vital signs (VS) to triage and prioritise medical urgency; however, it is not intended to support referral decisions [34–36]. In addition to the RETTS©, ambulance services had access to the web-based ADSS, a symptom-based tool used to support care needs assessment and provide self-care advice, during the study period. Originating from the Swedish national telephone triage service (1177), the ADSS is a symptom-based tool used nationwide to support licensed healthcare professionals in assessing care need and providing self-care advice. Its medical content is continuously reviewed and validated by a national editorial committee of expert physicians and nurses to ensure that the decision documents remain updated and aligned with national clinical guidelines [37].

### Data collection

The study cohort comprised all ambulance assignments categorised as self-care referrals between 1 January 2023, and 31 December 31 2023. However, owing to a data journal system update, data from one region were extracted between 31 August 2022 and 1 September 2023. Designated regional teams extracted the data by following approved confidentiality protocols. The dataset was delivered to the researchers in password-protected, encrypted files, and patient anonymity was maintained via pseudonymisation.

### Inclusion and exclusion process

The study population was identified through a systematic inclusion and exclusion process. Initially, all primary ambulance assignments were extracted (*n* = 88,283). In Exclusion Step 1, cases involving patient conveyance or referral to another level of care were removed (*n* = 81,131; 91.7%). Consequently, the study population consisted exclusively of patients who, following assessment by ambulance clinicians, were referred to self-care (self-care assignments, *n* = 7,152 (8.3%).

To ensure data quality and support model stability, Exclusion Step 2 was applied, resulting in the removal of an additional 700 assignments. Of these 700 assignments, the majority (*n* = 523) consisted of assignments where the caller was referred to self-care by the ambulance service more than once within 30 days. Excluding these assignments resulted in a dataset where each patient was represented at most once every 30 days. The remaining 177 excluded assignments were removed due to missing essential data—specifically, assignments where no triage was completed (ESS code 100, *n* = 146), cases with missing patient age (*n* = 10), or assignments falling outside the study scope (*n* = 21). The latter including EMCC Priority 4 assignments (*n* = 9), patients originating from other healthcare regions (*n* = 5), and cases in which patients were ultimately conveyed (*n* = 7) despite an initial self-care categorisation.

Following all exclusion steps, a total of 6,452 assignments were included in the final analysis.

### Study outcomes

The primary endpoint was subsequent SHC within 72 h of the initial self-care referral. This was analysed as a composite endpoint comprising ambulance recontacts, PHC visits, ED visits, and hospitalisations. Because individual patient care trajectories were not tracked, a single patient could appear in multiple outcome categories if more than one level of care was used during the 72‑hour period.

The secondary endpoint was all-cause mortality at 72 h and 30 days. The 30-day mortality measure was included to ensure comparability with the international emergency care literature and to provide an overview of the general prognosis of the study population.

### Data analysis

The total patient population was stratified by age into paediatric (aged < 18 years) and adult (aged ≥ 18 years) cohorts, reflecting fundamental differences in physiological VS and normative reference frameworks according to the RETTS© (see Additional file 1). However, where relevant, age was treated as a continuous variable to retain full data granularity and avoid information loss associated with categorical grouping. The dataset comprised patients aged < 1–103 years.

The following variables were included in the analysis: *Geographical location* (DeSO), classified into three urbanisation categories, rural, suburban and smaller urban areas, and central urban areas; *Distance to ED*, kilometres to the nearest hospital with an ED, calculated from the assignments’ documented DeSO centroid; *EMCC priority*, categorised as Priority 1, 2, or 3; *Time of day*, with three-time intervals (morning, 08:00–15:59; evening, 16:00–23:59; and night, 00:00–07:59); *On-scene time (min)*; *Age*; *Legal gender*; *Chief complaint* (ESS code) with the specific ESS code documented by the ambulance clinicians; *Vital signs* (VS), last documented value used; *Level of consciousness*, dichotomised as unimpaired (by documented Reaction Level Scale (RLS) RLS 1 or the Alert, Confusion, Verbal, Pain or Unresponsive (ACVPU) A scale) or impaired (RLS 2–8 or C, V, P, U); and *ADSS use*, documented use (Yes/No).

Missing data were classified as ‘ADSS not used’ (see ***Limitations***). The variables reflect the assignment circumstances that were available at the time of the self-care referral decision. Missing VS data were classified as normal (see ***Limitations***). The dataset comprised 91 different ESS codes, which were aggregated into 11 assessment categories (see Additional file 2).

### Statistical analysis

Descriptive statistics are presented as numbers (n), percentages (%), and median and interquartile range (IQR), where applicable, and the outcomes reported as proportions. Multilevel logistic regression models were used to estimate the probability of SHC and mortality. For all estimates, the results are presented as mean probabilities or odds ratios (OR) with 95% credibility interval (CI) defined by the 2.5 and 97.5 percentiles of the corresponding posterior distribution. As the goal was not to test narrow null hypotheses, nor to dichotomize effects into “true” or “false” effects based on whether estimates met some arbitrary threshold, but rather to updating prior beliefs in the light of the data at hand, evidence was not discarded merely because a posterior odds ratio’s credibility interval covered 1.0.

Prior distributions were informed by existing literature, research, and the authors’ collective clinical expertise, and set using an informal estimate-talk-estimate-approach, permitting the formal integration of prior knowledge, such as the expected increased probability of ED visits with older age.

Separate models were fit to model the probability of recontact or mortality for *any* SHC within 72 h and for each type of recontact (PHC visit, ambulance service recontact, ED visit, and hospitalisation) within 72 h, mortality within 72 h, and mortality within 30 days. Furthermore, for each outcome, separate models were fit for adults and children.

All models follow an identical general structure: a multilevel logistic regression model with regularising priors.

Letting $\:{y}_{i}$ denote the binary outcome for the *i*th patient, any one of the models can be expressed as

$$\:\begin{array}{cc}{y}_{i}&\sim\mathrm{B}\mathrm{e}\mathrm{r}\mathrm{n}\mathrm{o}\mathrm{u}\mathrm{l}\mathrm{l}\mathrm{i}\left({\theta}_{i}\right),\\\:\mathrm{l}\mathrm{o}\mathrm{g}\mathrm{i}\mathrm{t}\left({\theta}_{i}\right)&={\alpha}_{0}+{\alpha}_{c\left[i\right]}+{\mathbf{X}}_{i}^{{\top}}\boldsymbol{\beta},\\\:{\alpha}_{c}&\sim\mathrm{N}\mathrm{o}\mathrm{r}\mathrm{m}\mathrm{a}\mathrm{l}\left({\gamma}_{g\left[c\right]},\cdot\right)\end{array},$$

where $\:{\theta}_{i}$ is the probability of an SHC (or mortality), $\:{\alpha}_{0}$ the overall intercept, $\:{\alpha}_{c\left[i\right]}$ the varying intercept for the assessment code (ESS code) ‘*c*’ used for the *i*th patient, $\:{\gamma}_{g\left[c\right]}$ a group-level intercept for the assessment category *g* to which *c* belongs, and $\:{\mathbf{X}}_{i}$ a vector of standardised predictors for the *i*th patient with corresponding regression coefficients $\:\boldsymbol{\beta}$.

The predictors included in $\:{\mathbf{X}}_{i}$ were the patient’s legal gender, age, respiratory rate, oxygen saturation, heart rate, blood pressure, temperature, and consciousness level, as well as time of alarm (categorised as morning, evening, or night), distance to the nearest hospital, on-scene time, and ADSS use.

To reflect the authors’ prior understanding that the outcome is driven primarily by differences between assessment codes categories, rather than by the predictors, the method of Yanchenko et al. [48] was followed, and a shrinkage prior used on the model fit [49]. Rather than specifying independent priors, which have limited prior knowledge, a shared variance parameter $\:W$ was used, and the variance between the assessment code varying intercepts, assessment category group-level intercepts, and regression coefficients allocated using a Dirichlet prior. Within each component, the variance was distributed evenly. Specifically,

$$\:\begin{array}{cc}{\alpha}_{c}&\sim\mathrm{N}\mathrm{o}\mathrm{r}\mathrm{m}\mathrm{a}\mathrm{l}\left({\gamma}_{g\left[c\right]},{\varphi}_{\alpha}W\right),\\\:{\gamma}_{g}&\sim\mathrm{N}\mathrm{o}\mathrm{r}\mathrm{m}\mathrm{a}\mathrm{l}\left(0,{\varphi}_{\gamma}W\right),\\\:{\beta}_{k}&\sim\mathrm{N}\mathrm{o}\mathrm{r}\mathrm{m}\mathrm{a}\mathrm{l}\left(0,\frac{{\varphi}_{\beta}}{K}W\right),\\\:\boldsymbol{\varphi}&\sim\mathrm{D}\mathrm{i}\mathrm{r}\mathrm{i}\mathrm{c}\mathrm{h}\mathrm{l}\mathrm{e}\mathrm{t}\left({\xi}_{\alpha},{\xi}_{\gamma},{\xi}_{\beta}\right)\text{}\mathrm{with}\\&\:\boldsymbol{\varphi}=\left({\varphi}_{\alpha},{\varphi}_{\gamma},{\varphi}_{\beta}\right)\end{array},$$

which meant that only priors for the concentration parameters $\:\left({\xi}_{\alpha},{\xi}_{\gamma},{\xi}_{\beta}\right)$, the shared variance $\:W$, and the overall intercept $\:{\alpha}_{0}$ required specification.

For the concentration parameters, $\:\left({\xi}_{\alpha},{\xi}_{\gamma},{\xi}_{\beta}\right)=\left(\mathrm{3,2},1\right)$ was set to reflect the authors’ prior theories that half, one-third, and one-sixth of the variance should be allocated to the assessment code varying intercepts, assessment category group-level intercepts, and regression coefficients, respectively.

For the shared variance $\:W$, a prior that reflects the authors’ prior belief about the amount of variance explained by the model, as measured by the coefficient of determination $\:{R}^{2}$ was set [49]. Specifically, a generalised beta prime prior on $\:W$ that implies a prior on $\:{R}^{2}$ was used.

$$\begin{aligned}&W\sim\mathrm{G}\mathrm{e}\mathrm{n}\mathrm{e}\mathrm{r}\mathrm{a}\mathrm{l}\mathrm{i}\mathrm{s}\mathrm{e}\mathrm{d}\mathrm{B}\mathrm{e}\mathrm{t}\mathrm{a}\mathrm{P}\mathrm{r}\mathrm{i}\mathrm{m}\mathrm{e}\left(\mathrm{1,4},1,\frac{1}{\overline{y}\left(1-\overline{y}\right)}\right)\cr&\quad\stackrel{\approx}{\Rightarrow}{R}^{2}\sim\mathrm{B}\mathrm{e}\mathrm{t}\mathrm{a}\left(\mathrm{1,4}\right),\end{aligned}$$

where $\:\overline{y}$ is the mean of the outcome in the data. The parameters of the implied beta distribution were chosen to provide strong regularisation towards smaller values of $\:{R}^{2}$, which demonstrably works well when the model only captures a small portion of the total variance and in sparse high-dimensional settings [48].

Finally, although the same concentration parameters and prior for the shared variance across all models were used, the prior for the overall intercept $\:{\alpha}_{0}$ was separately chosen for each outcome using the aforementioned estimate-talk-estimate approach. This resulted in a value for the prior mean and an upper and lower bound between which the 95% prior predictive interval was required to be positioned. These values were used to set a normal prior for $\:{\alpha}_{0}$ with a mean equal to the prior mean and standard deviation, chosen such that the 2.5th and 97.5th percentiles of the normal distribution corresponded to the lower and upper bounds, respectively. The chosen bounds were 5–40% for any SHC and PHC visit, 5–20% for ambulance service recontact and ED visit, 10–30% for hospitalisation, 0–5% for 72-hour mortality, and 0–10% for 30-day mortality.

The model was fit in Stan (v. 2.36.0; [51]) using the cmdstanr interface (v. 0.9.0; [50]) for R (v. 4.4.3; [52]) (see Additional file 3). Model convergence was assessed using Stan’s inbuilt diagnostics, ensuring that all $\:\widehat{\mathrm{R}}$-values were < 1.01, effective sample sizes were reasonably large, and no indications of divergent transitions were noted. Posterior predictive checks were performed to evaluate the model’s performance by simulating outcomes for a new, identical cohort and comparing the predicted and observed values, considering qualitative agreement as evidence that the model is at least superficially compatible with the data (see Additional files 4 and 5).

## Results

### Study cohort

**Fig. 1:**
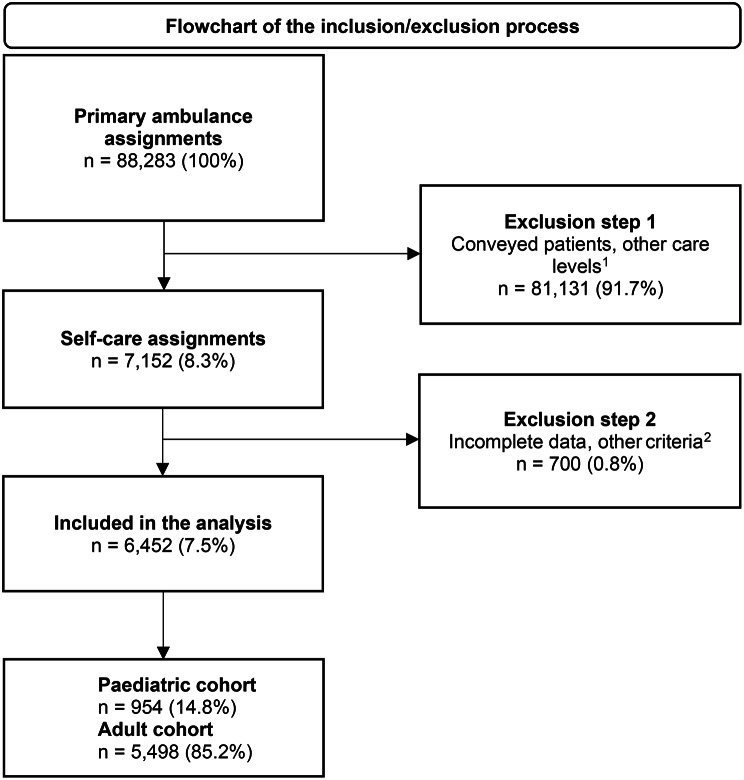
Inclusion and exclusion process: Self-care referral during 2023, in three Swedish regions. (1) Patients referred to PHC or other non-conveyance assignments. (2) (a) Conveyed patients, *n* = 7 (> 0.1%); (b) EMCC Priority 4, *n* = 9 (< 0.1%); (c) Frequent callers, *n* = 523 (7.3%); (d) Other regions, *n* = 5 (< 0.1%); (e) No triage (ESS 100) *n* = 146 (> 0.01%); and (f) No age, *n* = 10 (< 0.01%)

After the inclusion and exclusion process, a total of 6,452 assignments were included in the analysis (Fig. 1). The final cohort was divided into an adult population of 5,498 patients (85,2%) and a paediatric population of 954 patients (14,8%).

The results are structured as follows: Part 1 provides a purely descriptive statistics of the study cohort and outcomes, while Part 2 presents the statistical analysis of the predictors and the predicted probabilities for SHC and mortality.

### Part 1: Descriptive statistics

#### Paediatric cohort – SHC and mortality

The paediatric cohort comprised 954 patients (Table 1). Of these, 23.7% sought one or more SHC < 72 h after the initial on-scene assessment and self-care referral, including PHC visits (60%), ambulance service recontact (15%), ED visits (33%), and hospitalisation (10%). More than half of the assignments were Priority 1 EMCC-alerts. Most of the assignments were in an urban area during the evening. The three most common documented SHCs were respiratory, infection, or medical symptoms. No mortality was observed.^1^

**Table 1:** Characteristics and SHC within 72 h among children (aged < 18 years) referred to self-care

Variable, *n* (%)	No SHC^a^	SHC	PHC visit^b^	Recon amb^c^	ED visit^d^	Hospitalised
	728 (76.3%)	226 (23.7%)	136 (14.3%)	34 (3.6%)	76 (8%)	22 (2.3%)
**Age (years), median (IQR)**						
	5 (2–13)	3 (1–8)	3 (1–6.2)	4 (1–13.5)	2 (1–8.2)	2 (1–8.5)
**Legal gender, n (%)**						
Male	383 (53.2%)	109 (48.2%)	70 (51.5%)	13 (38.2%)	36 (47.4%)	12 (54.5%)
Female	337 (46.8%)	117 (51.8%)	66 (48.5%)	21 (61.8%)	40 (52.6%)	10 (45.5%)
**Geographical location, category, n (%)**						
Urban	458 (64%)	153 (70.2%)	90 (69.2%)	17 (54.8%)	54 (71.1%)	14 (63.6%)
Suburban	113 (15.8%)	27 (12.4%)	17 (13.1%)	5 (16.1%)	8 (10.5%)	3 (13.6%)
Rural	145 (20.3%)	38 (17.4%)	23 (17.7%)	9 (29%)	14 (18.4%)	5 (22.7%)
**Time of day, n (%)**						
Morning (08:00–15:59)	181 (24.9%)	48 (21.2%)	24 (17.6%)	10 (29.4%)	18 (23.7%)	4 (18.2%)
Evening (16:00–23:59)	410 (56.3%)	109 (48.2%)	71 (52.2%)	13 (38.2%)	30 (39.5%)	11 (50%)
Night (00:00–07:59)	137 (18.8%)	69 (30.5%)	41 (30.1%)	11 (32.4%)	28 (36.8%)	7 (31.8%)
**Hospital distance (km), median (IQR)**						
	24 (4.8–35.5)	23.5 (5.1–34.6)	25.6 (9.3–38.5)	17.3 (3.3–29.8)	16.1 (5–31.4)	15.7 (5.1–29.7)
**Time on scene (min), median (IQR)**						
	23.8 (16–32.6)	25.4 (18.1–36.1)	25.8 (18.7–36)	25.8 (18.5–41.4)	25.2 (17.8–37.2)	22.9 (17.3–36.9)
**Vital signs, median (IQR)**						
Respiratory rate (breaths/min)	20 (18–26)	24 (20–30)	24 (20–28)	21 (19–30)	25.5 (22–32)	28 (20–36)
Oxygen saturation (%)	99 (98–99)	99 (97–100)	99 (98–100)	99 (97–99)	98 (97–100)	98 (98–99)
Heart rate (beats/min)	110 (92.8–132)	124 (107.5–144)	123.5 (105.2–141)	122.5 (104.5–150)	135.5 (118.8–150.2)	142.5 (120–156.2)
Blood pressure (mmHg)	117 (110–125)	120 (110–123)	120 (110–124)	115.5 (108.5–126.2)	115 (108.8–120)	115 (106.8–125.2)
Temperature (°C)	37.1 (36.7–37.9)	37.6 (36.9–38.6)	37.7 (36.9–38.6)	37.3 (37–37.7)	37.7 (36.9–38.8)	37.9 (37–38.8)
**Consciousness, n (%)**						
Impaired (RLS 2–8/CVPU)	8 (1.5%)	3 (1.7%)	2 (1.9%)	0 (0%)	1 (1.8%)	1 (5.6%)
Unimpaired (RLS 1/Alert)	535 (98.5%)	169 (98.3%)	102 (98.1%)	25 (100%)	55 (98.2%)	17 (94.4%)
**ADSS**^**e**^ **use, n (%)**						
	288 (67.8%)	93 (69.4%)	68 (73.9%)	5 (41.7%)	30 (60%)	7 (46.7%)
**On-scene assessment category, n (%)**						
Circulatory symptoms	13 (1.8%)	1 (0.4%)	0 (0%)	0 (0%)	1 (1.3%)	0 (0%)
Eye, ear, nose, throat symptoms	18 (2.5%)	2 (0.9%)	2 (1.5%)	0 (0%)	0 (0%)	0 (0%)
Gynaecology, urology symptoms	3 (0.4%)	0 (0%)	0 (0%)	0 (0%)	0 (0%)	0 (0%)
Infection symptoms	87 (12%)	44 (19.5%)	28 (20.6%)	5 (14.7%)	16 (21.1%)	6 (27.3%)
Medical symptoms	129 (17.7%)	35 (15.5%)	22 (16.2%)	4 (11.8%)	10 (13.2%)	1 (4.5%)
Neurology symptoms	85 (11.7%)	23 (10.2%)	14 (10.3%)	7 (20.6%)	6 (7.9%)	6 (27.3%)
Orthopaedic symptoms	4 (0.5%)	3 (1.3%)	2 (1.5%)	0 (0%)	1 (1.3%)	0 (0%)
Psychiatric symptoms	32 (4.4%)	4 (1.8%)	2 (1.5%)	1 (2.9%)	1 (1.3%)	0 (0%)
Respiratory symptoms	164 (22.5%)	65 (28.8%)	43 (31.6%)	9 (26.5%)	20 (26.3%)	4 (18.2%)
Surgical symptoms	67 (9.2%)	24 (10.6%)	10 (7.4%)	1 (2.9%)	13 (17.1%)	4 (18.2%)
Trauma	126 (17.3%)	25 (11.1%)	13 (9.6%)	7 (20.6%)	8 (10.5%)	1 (4.5%)
**EMCC**^**f**^ **priority, n (%)**						
Priority 1	368 (50.5%)	125 (55.3%)	415 (50.7%)	78 (57.4%)	473 (51.4%)	20 (58.8%)
Priority 2	348 (47.8%)	100 (44.2%)	391 (47.8%)	57 (41.9%)	434 (47.2%)	14 (41.2%)
Priority 3	12 (1.6%)	1 (0.4%)	12 (1.5%)	1 (0.7%)	13 (1.4%)	0 (0%)

^a^Patient did not have an SHC

^b^Patient visited the PHC

^c^Patient recontacted the ambulance service

^d^Patient visited the ED

^e^If ambulance clinicians used the web-based ADSS

^f^Emergency medical communication centre

#### Adult cohort – SHC

The adult cohort comprised 5,498 patients (Table 2). Of these, 29.2% sought one or more SHC within 72 h after the initial on-scene assessment and self-care referral, including PHC visits (55%), ambulance service recontact (40%), ED visits (30%), and hospitalisation (15%). Half of these assignments were Priority 2 EMCC-alerts, and one-third were Priority 1 EMCC-alerts. Most of the assignments were in an urban area during the evening. The three most common SHCs documented were medical, surgical, or neurological symptoms.

**Table 2:** Characteristics and SHC within 72 h among adults

Variable	No SHC^a^	SHC	PHC visit^b^	Recon amb^c^	ED visit^d^	Hospitalised
n (%)	3894 (70.8%)	1604 (29.2%)	895 (16.3%)	622 (11.3%)	490 (8.9%)	240 (4.4%)
**Age (years)**,** median (IQR)**						
	69 (42–82)	73 (51–83)	75 (54–83.5)	76 (60–85)	73 (49–82)	78 (65–85)
**Legal sex**,** n (%)**						
Male	1761 (45.3%)	759 (47.3%)	420 (46.9%)	301 (48.4%)	231 (47.1%)	113 (47.1%)
Female	2128 (54.7%)	845 (52.7%)	475 (53.1%)	321 (51.6%)	259 (52.9%)	127 (52.9%)
**Geographical location category**,** n (%)**						
Urban	2436 (63.2%)	1020 (64.4%)	556 (63.1%)	410 (66.6%)	314 (65.1%)	150 (63%)
Suburban	637 (16.5%)	255 (16.1%)	150 (17%)	97 (15.7%)	77 (16%)	36 (15.1%)
Rural	780 (20.2%)	308 (19.5%)	175 (19.9%)	109 (17.7%)	91 (18.9%)	52 (21.8%)
**Time of day**,** n (%)**						
Morning (08:00–15:59)	1417 (36.4%)	552 (34.4%)	303 (33.9%)	218 (35%)	173 (35.3%)	88 (36.7%)
Evening (16:00–23:59)	1587 (40.8%)	655 (40.8%)	372 (41.6%)	256 (41.2%)	197 (40.2%)	94 (39.2%)
Night (00:00–07:59)	890 (22.9%)	397 (24.8%)	220 (24.6%)	148 (23.8%)	120 (24.5%)	58 (24.2%)
**Hospital distance (km)**,** median (IQR)**						
	20.7 (3.1–34.5)	23.4 (4.4–35.4)	25.1 (5.4–35.8)	20.3 (3.6–35.3)	22.7 (4.6–35.3)	25.1 (7.5–38.5)
**Time on scene (min)**,** median (IQR)**						
	25.4 (18.4–34.7)	27.4 (19.7–37.6)	27 (19.5–37.6)	28.6 (20.2–38.7)	29.3 (20.2–39.4)	31.8 (23–42.7)
**Vital signs**,** median (IQR)**						
Respiratory rate (breaths/min)	18 (16–20)	18 (16–20)	18 (16–20)	18 (16–20)	18 (16–20)	18 (16–20)
Oxygen saturation (%)	97 (96–99)	97 (96–99)	98 (96–99)	97 (95–98)	98 (96–99)	97 (96–98)
Heart rate (beats/min)	83 (74–94)	82 (73–95)	81.5 (73–95)	82 (73–94)	83 (72–97.2)	85 (75–98.5)
Blood pressure (mmHg)	135 (120–150)	137 (120–150)	140 (120–154)	135 (120–150)	140 (124–155)	140 (120–153)
Temperature (°C)	36.8 (36.5–37.2)	36.9 (36.5–37.2)	36.9 (36.5–37.2)	36.8 (36.5–37.3)	36.9 (36.6–37.3)	37 (36.6–37.4)
**Consciousness**,** n (%)**						
Impaired (RLS 2–8/CVPU)	39 (1.2%)	17 (1.3%)	11 (1.5%)	5 (0.9%)	4 (1%)	4 (2%)
Unimpaired (RLS 1/Alert)	3313 (98.8%)	1338 (98.7%)	746 (98.5%)	525 (99.1%)	397 (99%)	193 (98%)
**ADSS**^**e**^ **use**,** n (%)**						
	1247 (32%)	633 (39.5%)	398 (44.5%)	245 (39.4%)	263 (53.7%)	147 (61.3%)
**On-scene assessment category**,** n (%)**						
Circulatory symptoms	485 (12.5%)	154 (9.6%)	83 (9.3%)	57 (9.2%)	45 (9.2%)	17 (7.1%)
Eye, ear, nose, throat symptoms	21 (0.5%)	12 (0.7%)	8 (0.9%)	4 (0.6%)	1 (0.2%)	1 (0.4%)
Gynaecology, urology symptoms	47 (1.2%)	22 (1.4%)	14 (1.6%)	6 (1%)	6 (1.2%)	2 (0.8%)
Infection symptoms	290 (7.4%)	146 (9.1%)	79 (8.8%)	62 (10%)	47 (9.6%)	35 (14.6%)
Medical symptoms	840 (21.6%)	265 (16.5%)	148 (16.5%)	102 (16.4%)	70 (14.3%)	39 (16.2%)
Neurology symptoms	552 (14.2%)	188 (11.7%)	115 (12.8%)	70 (11.3%)	52 (10.6%)	26 (10.8%)
Orthopaedic symptoms	165 (4.2%)	134 (8.4%)	95 (10.6%)	45 (7.2%)	42 (8.6%)	18 (7.5%)
Psychiatric symptoms	241 (6.2%)	90 (5.6%)	40 (4.5%)	38 (6.1%)	21 (4.3%)	11 (4.6%)
Respiratory symptoms	279 (7.2%)	172 (10.7%)	91 (10.2%)	85 (13.7%)	43 (8.8%)	26 (10.8%)
Surgical symptoms	517 (13.3%)	246 (15.3%)	122 (13.6%)	96 (15.4%)	100 (20.4%)	40 (16.7%)
Trauma	457 (11.7%)	175 (10.9%)	100 (11.2%)	57 (9.2%)	63 (12.9%)	25 (10.4%)
**EMCC**^**f**^ **Priority**,** n (%)**						
Priority 1	1472 (37.8%)	537 (33.5%)	290 (32.4%)	204 (32.8%)	160 (32.7%)	82 (34.2%)
Priority 2	2170 (55.7%)	912 (56.9%)	503 (56.2%)	362 (58.2%)	282 (57.6%)	138 (57.5%)
Priority 3	252 (6.5%)	155 (9.7%)	102 (11.4%)	56 (9%)	48 (9.8%)	20 (8.3%)

^a^Patient did not have an SHC

^b^Patient visited the PHC

^c^Patient recontacted the ambulance service

^d^Patient visited the ED

^e^If ambulance clinicians used the web-based ADSS

^f^Emergency medical communication centre

#### Adult cohort – 72-hour and 30-day mortality

In total, 24 (0.4%) patients, with a median age of 87.5 years versus 69 years for those with no SHC, died within 72 h (Tables 2 and 3). Approximately 50% had impaired consciousness during the initial on-scene assessment. In addition, these patients’ VS differed from the other outcomes: increased breathing rate, 20 breaths/min (18–23); decreased saturation, 91% (85–95%); and increased heartrate, 100 beats/min (82–110). These assignments had the longest on-scene time, with a median of 34.5 min (26–57.2). Most of the assignments were in an urban area during the evening, and most (66%) were Priority 1 EMCC-alerts. The patients initially sought ambulance services for medical, neurological, psychiatric, orthopaedic, respiratory, and surgical symptoms. Approximately 47% of the patients who died sought one or more SHC within 72 h for mostly medical, respiratory, or neurological symptoms.

**Table 3:** Characteristics and SHC and mortality 72 h/30 days after self-care referral among adults (*n* = 5,498)

Variable	SHC^a^	Mortality < 72 h^b^	Mortality < 30 days^c^
**n (%)**	1604 (29.2%)	24 (0.4%)	101 (1.8%)
**Age (years)**,** median (IQR)**			
	73 (51–83)	87.5 (80–93.2)	86 (79–91)
**Legal sex**,** n (%)**			
Male	759 (47.3%)	11 (45.8%)	45 (44.6%)
Female	845 (52.7%)	13 (54.2%)	56 (55.4%)
**Geographical location category**,** n (%)**			
Urban	1020 (64.4%)	15 (62.5%)	59 (58.4%)
Suburban	255 (16.1%)	5 (20.8%)	20 (19.8%)
Rural	308 (19.5%)	4 (16.7%)	22 (21.8%)
**Time of day**,** n (%)**			
Morning (08:00–15:59)	552 (34.4%)	9 (37.5%)	44 (43.6%)
Evening (16:00–23:59)	655 (40.8%)	12 (50%)	38 (37.6%)
Night (00:00–07:59)	397 (24.8%)	3 (12.5%)	19 (18.8%)
**Hospital distance (km)**,** median (IQR)**			
	23.4 (4.4–35.4)	27 (3.7–38.4)	25.1 (5–39)
**Time on scene (min)**,** median (IQR)**			
	27.4 (19.7–37.6)	34.5 (26–57.2)	32.2 (22.7–45.9)
**Vital signs**,** (median (IQR)**			
Respiratory rate (breaths/min)	18 (16–20)	20 (18–23)	18 (17–20)
Oxygen saturation (%)	97 (96–99)	91 (85–95)	95 (92–98)
Heart rate (beats/min)	82 (73–95)	100 (82–110)	88 (76–100)
Blood pressure (mmHg)	137 (120–150)	120 (102.5–131.5)	120 (109–140)
Temperature (°C)	36.9 (36.5–37.2)	36.6 (36.4–37.2)	36.7 (36.4–37.2)
**Consciousness**,** n (%)**			
Impaired (RLS 2–8/CVPU)	17 (1.3%)	10 (47.6%)	11 (13.1%)
Unimpaired (RLS 1/Alert)	1338 (98.7%)	11 (52.4%)	73 (86.9%)
**ADSS**^**d**^ **use**,** n (%)**			
	633 (39.5%)	5 (20.8%)	41 (40.6%)
**On-scene assessment category**,** n (%)**			
Circulatory symptoms	154 (9.6%)	0 (0%)	4 (4%)
Eye, ear, nose, throat symptoms	12 (0.7%)	0 (0%)	2 (2%)
Gynaecology, urology symptoms	22 (1.4%)	0 (0%)	1 (1%)
Infection symptoms	146 (9.1%)	2 (8.3%)	7 (6.9%)
Medical symptoms	265 (16.5%)	8 (33.3%)	33 (32.7%)
Neurology symptoms	188 (11.7%)	3 (12.5%)	14 (13.9%)
Orthopaedic symptoms	134 (8.4%)	1 (4.2%)	3 (3%)
Psychiatric symptoms	90 (5.6%)	2 (8.3%)	6 (5.9%)
Respiratory symptoms	172 (10.7%)	6 (25%)	19 (18.8%)
Surgical symptoms	246 (15.3%)	1 (4.2%)	8 (7.9%)
Trauma	175 (10.9%)	1 (4.2%)	4 (4%)
**EMCC**^**e**^ **priority**,** n (%)**			
Priority 1	537 (33.5%)	16 (66.7%)	53 (52.5%)
Priority 2	912 (56.9%)	8 (33.3%)	41 (40.6%)
Priority 3	155 (9.7%)	0 (0%)	7 (6.9%)

^a^Patient had an SHC

^b^Patient died within 72 h after self-care referral

^c^Patient died within 30 days after self-care referral

^d^If ambulance clinicians used the web-based ADSS

^e^Emergency medical communication centre

In total, 101 (1.8%) patients, with a median age of 86 years versus 69 years for those with no SHC, died within 30 days (Table 2). Most of the assignments were in an urban area during daytime. Approximately 52% were Priority 1 EMCC-alerts. Medical, respiratory, or neurological symptoms were most common. Approximately half of these patients sought SHC for mostly medical, neurological, or surgical symptoms. However, nearly all assessment categories were observed except for gynaecology and urology symptoms.

### Part 2: Predictors and predicted probability of SHC and mortality within 72 h and 30 days

#### Predicted probability of SHC in children

**Fig. 2:**
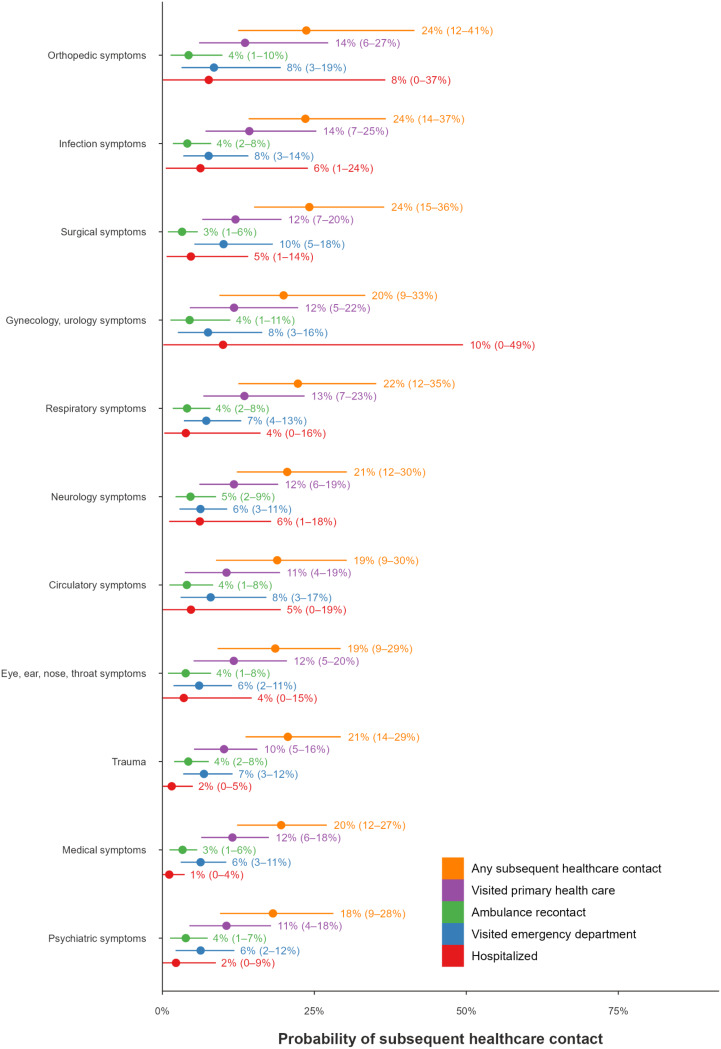
Predicted probabilities of subsequent healthcare contact (SHC) by assessment category for children. For each predictor, estimates are shown for the five outcomes, in the following order: any SHC, primary healthcare centre visit, ambulance service recontact, emergency department visit, and hospitalisation, all occurring within 72 h. Points indicate posterior means, and horizontal lines represent 95% credibility intervals

**Fig. 3:**
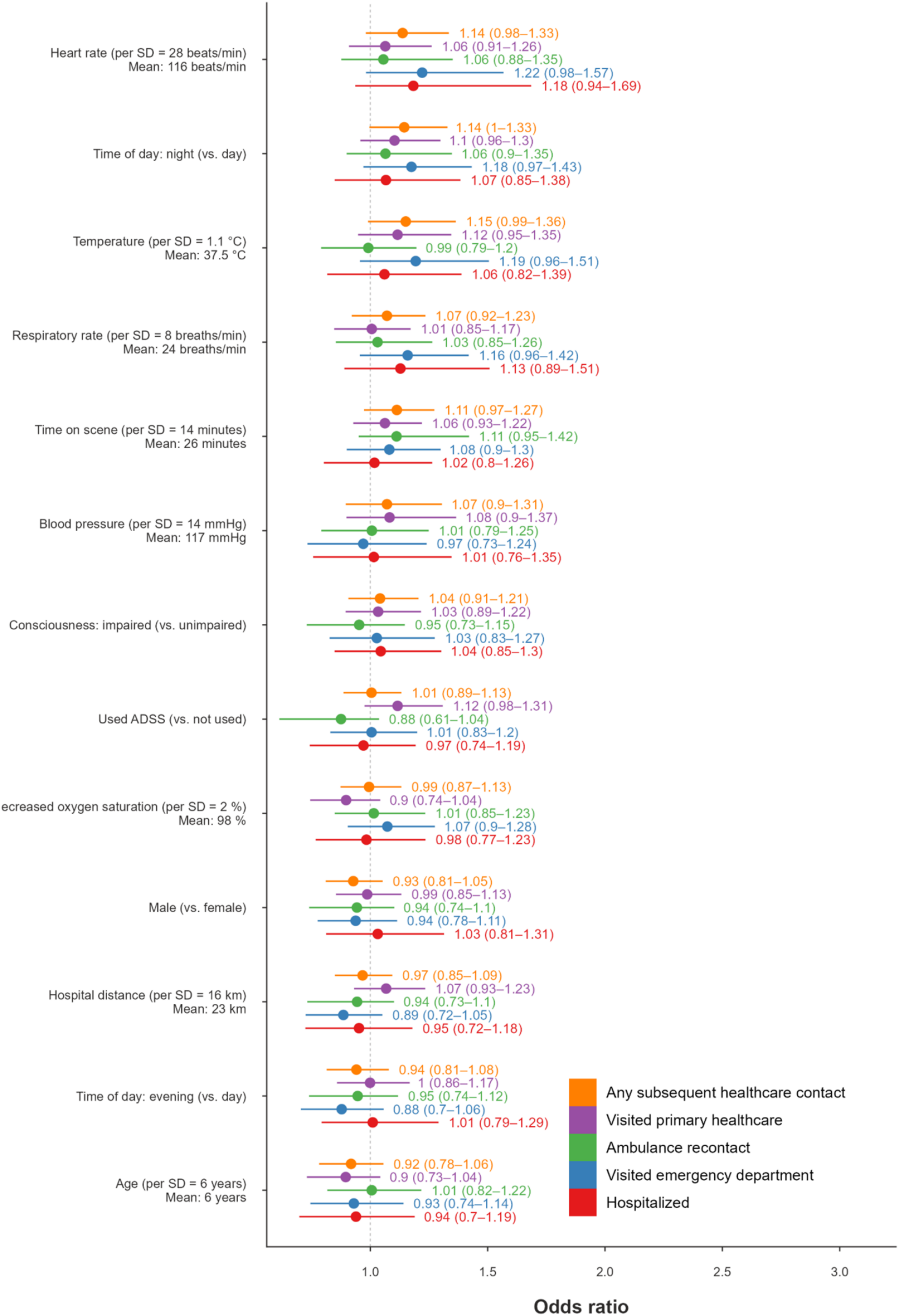
Posterior odds ratios of subsequent healthcare contact (SHC) for children. For each predictor, estimates are shown for the five outcomes, in the following order: any SHC, primary healthcare centre visit, ambulance service recontact, emergency department visit, and hospitalisation, all occurring within 72 h. Points indicate posterior means, and horizontal lines represent 95% credibility intervals

The assessment categories with the highest estimated mean predicted probability (PP) of SHC were orthopaedic (mean PP = 24%, 95% CI: 12–41%), infection (mean PP = 24%, 95% CI: 14–37%), and surgical (mean PP = 24%, 95% CI: 15–36%) symptoms. The assessment categories with the lowest estimated mean probability of SHC were psychiatric (mean PP = 18%, 95% CI: 9–28%) and medical (mean PP = 20%, 95% CI: 12–27%) symptoms. The predictors with the highest mean posterior odds ratio for SHC were increased temperature (OR 1.15, 95% CI: 0.99–1.36), assessments during the night (OR 1.14, 95% CI: 1.00–1.33) and increased heart rate (OR 1.14, 95% CI: 0.98–1.33) (Figs. 2 and 3).

#### Predicted probability of SHC in adults

**Fig. 4:**
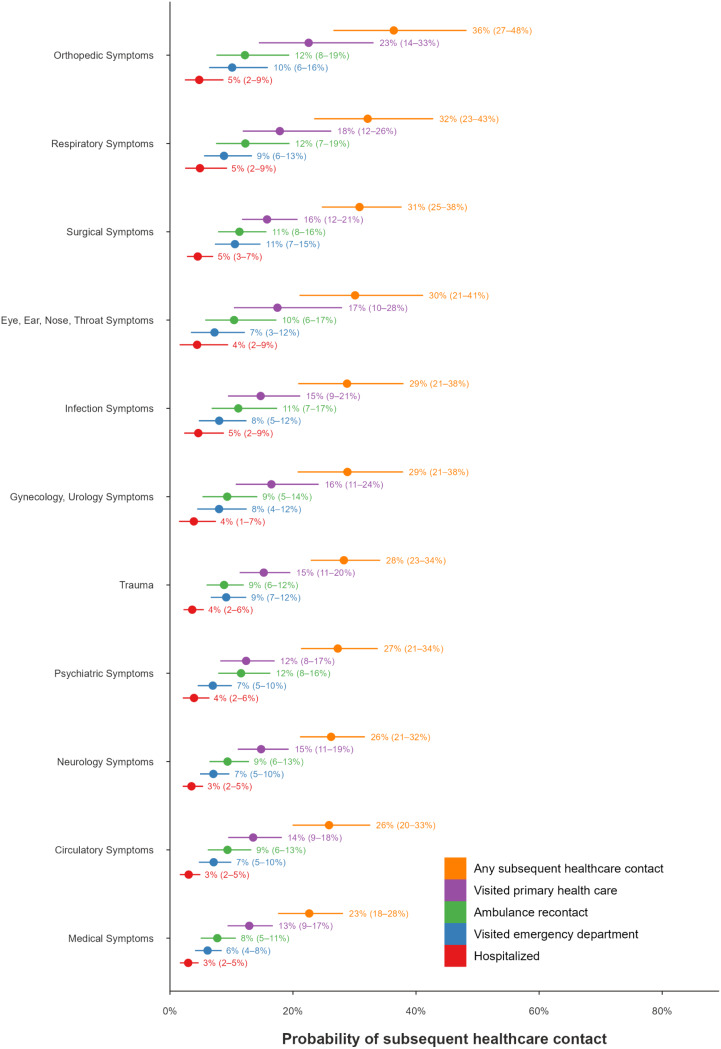
Predicted probabilities of subsequent healthcare contact (SHC) by assessment category for adults. For each predictor, estimates are shown for the five outcomes, in the following order: any SHC, primary healthcare centre visit, ambulance service recontact, emergency department visit, and hospitalisation, all occurring within 72 h. Points indicate posterior means, and horizontal lines represent 95% credibility intervals

**Fig. 5:**
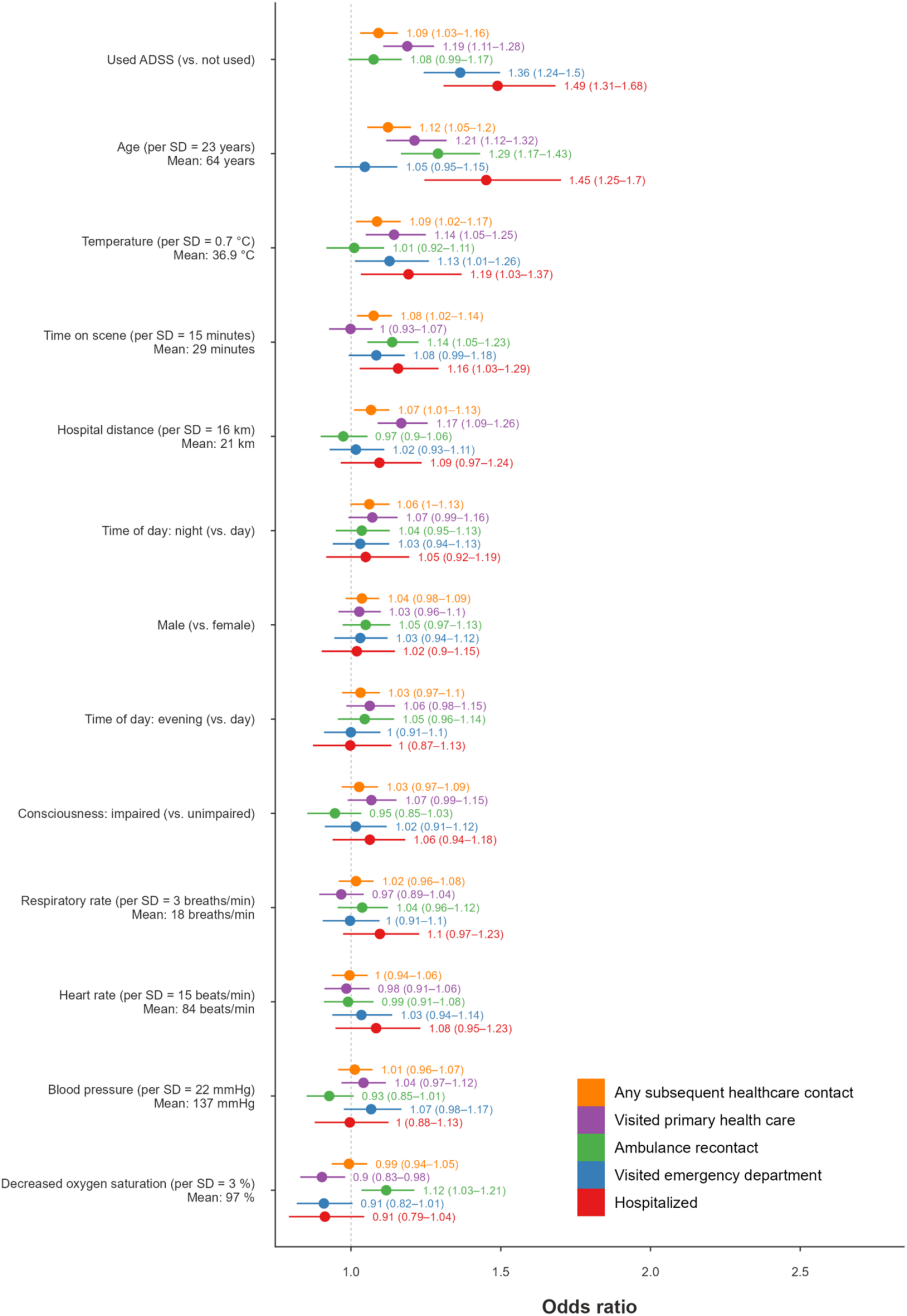
Posterior odds ratios of subsequent healthcare contact (SHC) for adults. For each predictor, estimates are shown for the five outcomes, in the following order: any SHC, primary healthcare centre visit, ambulance service recontact, emergency department visit, and hospitalisation, all occurring within 72 h. Points indicate posterior means, and horizontal lines represent 95% credibility intervals

The assessment categories with the highest estimated mean PP of SHC were orthopaedic (mean PP = 36%, 95% CI: 27–48%), respiratory and infection (mean PP = 33%, 95% CI: 24–43%), and surgical (mean PP = 31%, 95% CI: 25–38%) symptoms. The assessment categories with the lowest estimated mean PP of SHC were medical (mean PP = 23%, 95% CI: 18–28%), circulatory (mean PP = 26%, 95% CI: 20–33%), and neurological (mean PP = 26%, 95% CI: 21–32%) symptoms. The predictors with the highest mean posterior odds ratio for SHC were increased age (OR 1.12, 95% CI: 1.05–1.20), documented use of ADSS (OR 1.09, 95% CI: 1.03–1.16), increased temperature (OR 1.09, 95% CI: 1.02–1.17), time on scene (OR 1.08, 95% CI: 1.02–1.14) and hospital distance (OR 1.07, 95% CI: 1.01–1.13). (Figures 4 and 5).

#### Predicted probability of mortality in adults

The predictors with the highest mean posterior odds ratio for mortality within 72 h were increased age (OR 2.93, 95% CI: 1.37–6.59), increased heart rate (OR 1.77, 95% CI: 1.11–2.82), impaired consciousness (OR 1.47, 95% CI: 1.28–1.70) and decreased oxygen saturation (OR 1.39, 95% CI: 1.15–1.70).

The predictors with the highest mean posterior odds ratio for 30-day mortality were increased age (OR 3.45, 95% CI: 2.36–5.02), increased heart rate (OR 1.33, 95% CI: 1.07–1.63), documented use of ADSS (OR 1.25, 95% CI: 1.01–1.53), decreased oxygen saturation (OR 1.24, 95% CI: 1.08–1.42), impaired consciousness (OR 1.22, 95% CI: 1.10–1.35) and increased respiratory rate (OR 1.22, 95% CI: 1.04–1.42). The predictors with the lowest mean posterior odds ratio for 30-day mortality was increased blood pressure (OR 0.67, 95% CI: 0.54–0.83) (see Additional file 6).

## Discussion

### Overview of study findings and systemic challenges

To the authors’ knowledge, this study is the first to explicitly explore patient safety and predictors of SHC, after ambulance clinician self-care referrals. Hence, this study provides novel evidence to inform ambulance service-related clinical guidelines and decision-support systems. Overall, self-care referral decisions are generally safe in terms of life-threatening outcomes, with an observed low overall mortality rate. In addition, the mortality data in this study may include patients in palliative and end-of-life care. The high frequency of SHCs, however, presents a dual perspective that warrants a nuanced interpretation beyond simple clinical outcome measures. On one hand, SHC may reflect that safety-netting advice successfully guided patients to an appropriate level of care. On the other hand, they may indicate a failure to provide a definitive or legitimising care solution during the initial encounter. As elaborated below, these subsequent contacts may represent patients’ attempts to seek reassurance in response to unresolved distress or insufficient information provided by ambulance clinicians [46], rather than reflecting an intended or effective outcome of the safety-netting process.

### Navigating uncertainty: protocol-driven support and the vulnerability of ageing

The ADSS was implemented as a response to support the ambulance clinicians to refer patients to appropriate levels of care, assess care needs, and provide self-care advice. This study found that it increased the probability for SHC and hospitalisation, which may appear counter intuitive. The implementation of ADSS highlights the ambulance service’s shift towards an increasingly protocol-driven organisation. Lederman et al. reported that the ambulance clinicians expressed the need for adequate assessment systems to support them in the assessment of non-urgent symptoms [38]. Johansson et al. found a significant protocol-practice gap, revealing that only one-third (34%) of ambulance clinicians adhere to established guidelines, assessment tools, and mandatory patient information required for referrals [39]. However, the underlying reasons for this low adherence remain unexplored.

The present study revealed that approximately 90% of assignments were EMCC-categorised as Priority 1 or Priority 2, yet ultimately resulting in self-care referrals. The initial EMCC prioritisation reflects a safety-first approach in the early phase of an evolving illness, as described by Silverston’s Model of Illness [40], wherein clinical assessment can be considered as a snapshot of a dynamic process with three critical stages: Point A (initial assessment with high uncertainty), Point B (the window of opportunity where specific signs emerge), and Point C (critical physiological instability). The present study findings indicated normal VS during on-scene assessment (Point A), rendering it difficult for ambulance clinicians to discriminate between a self-limiting ‘blue line’ and a life-threatening ‘red line’ condition, as contrair clinical findings may not yet have developed.

Forsgärde et al. noted that this uncertainty is exacerbated in older persons, considering age-related physiological changes such as impaired function in respiratory, circulatory, and temperature compensation systems which complexify their clinical presentation [41]. In addition, ageing affects disease and injury handling abilities. Frailty in older persons is associated with physical decline and increased vulnerability. The present study indicates that ADSS use may contribute to enhanced patient safety by facilitating SHC at Point B, when the ‘red line’ becomes distinguishable, albeit before life-threatening complications arise. Furthermore, the study findings indicate that increased age is a predictor of nearly all SHCs and hospitalisations after self-care referrals, consistent with that reported by Paulin et al. that older age was common predictor of many events [42], likely reflecting increased care needs and probability of planned appointments, as well as the complexity of assessing older patients.

In this context, it is important to recognise that for the oldest age groups, where mortality was most common, death does not necessarily represent an adverse event. Instead, it may reflect the natural progression of frailty or existing limitations in care. For some of these patients, the decision to recommend self-care or non-conveyance may have been consistent with palliative care goals. However, the higher mortality observed among older patients may also partly reflect adverse events. Holmberg et al. demonstrated that ambulance clinicians risk perceiving older, frail, and vulnerable patients as a homogeneous group with an impaired decision-making capacity [43], leading to the simplification of patients’ experiences and withholding of information. This professional simplification may stem from a problematic assumption that certain patients seek care unnecessarily. Such perceptions can manifest in degrading clinical encounters in which patients feel dismissed; in extreme cases, being told that their call was ‘unnecessary’ can become a traumatic final memory for patients who subsequently deteriorate [3], potentially contributing to their SHC. Loor et al. examined the reasons for post-referral SHC [19], revealing that most patient-related reasons were illness-related, such as disease progression or recurrence/exacerbation. In 25% of cases, psychiatric disorders, substance abuse, or concerns expressed by patients and their significant others predominated. Approximately 20% of SHCs were associated with professional factors, including requests for reassessment by other care providers or misdiagnosis. The present study results suggest that during the on-scene assessment, the patient remains within the ‘blue line’, as indicated by normal VS. Moreover, the ADSS may capture a snapshot of this dynamic illness process, supporting ambulance clinicians in advising patients on the recognition of ‘red line’ symptoms. This may further explain the increased likelihood of SHC driven by ageing-related factors.

### Systemic factors, inequality, and decision-making complexity

The study findings revealed systemic and operational factors that increased the likelihood of SHC, highlighting areas where current practices may create inequality and expose risk. Specifically, longer on-scene time, longer distance to hospital, and ADSS use were all independently associated with a higher likelihood of SHC, even after adjusting for patient characteristics. This suggests complexity of the assessments and may reflect ambulance clinicians’ need for additional decision-making support: a finding supported by other studies [37, 44]. Knowles et al. observed that these outcomes were shaped by organisational culture and team composition; specifically, a leadership that perceives referrals primarily through the lens of risk, or teams with less clinical experience, may struggle to provide the reassurance necessary for safe care transitions [45]. Pauline et al. further highlighted the importance of team composition and education level [42]: lower education was associated with a higher risk of subsequent events. When self-care referrals occur despite ambulance clinicians’ longer on-scene time, SHC suggests that the initial decision was complex.

Furthermore, the association between a longer distance to the nearest ED and a higher likelihood of SHC raises important equity concerns. The study results demonstrate that an increased distance to nearest ED increased the likelihood of SHC, consistent with the findings of Paulin et al. [17], who observed that geographic location and distance to ED are key determinants of SHC, and with those of Jensen et al. [10], who reported that patients in rural areas were more likely to be referred, thus raising questions on the influence of geographic location on clinicians’ referral decision-making. Granlund et al. [44] emphasised that in rural areas PHC were less accessible, which may affect referral decisions. These systemic variability challenges health equity and risks neglecting the patient’s perspective.

Lederman et al. [46] highlights that a non-conveyance decision involves a profound transfer of responsibility. If a patient is left with ‘missing answers’ while experiencing existential fear, the burden of self-care may exceed their perceived ability to manage, leading to unnecessary distress and a subsequent search for security within the healthcare system. The clinical encounter becomes particularly sensitive when ambulance clinicians’ nuanced understanding of urgency is overshadowed by a judgmental attitude or the assumption that a call is ‘unnecessary’, especially given that patients often seek care in good faith to manage intolerable circumstances [5]. This suffering is further exacerbated when patients feel dismissed or encounter a lack of empathy, which not only jeopardises patient safety but also undermines their dignity during a period of acute vulnerability [3].

Finally, the link between ADSS use and increased SHCs may indicate that the systemic challenges in managing clinical complexity. Ambulance clinicians often seek reassurance in complex assessments [37, 44], and the present study findings indicate an increased likelihood of SHC when ADSS is used. This suggests that ambulance clinicians experience uncertainty in these situations, and ADSS reliance may reinforce decisions favouring self-care referrals in complex cases, indicating a potential limitation of the system. However, rather than interpret it as a system failure, further research is needed to understand ADSS use during real-time patient encounters.

### Systemic inefficiency and the orthopaedic challenge

In addition to Silverston’s ‘blue line’ and ‘red line’ conditions [39], the present study findings suggest the existence of a ‘yellow line’, especially for orthopaedic symptoms (37% SHC; 4.4% hospitalisation). Patients with such symptoms do not present acute risks at Point A, as indicated by normal VS, yet their condition (e.g., back pain) is not self-limiting. Vella et al. noted that knowledge regarding the safety of non-traumatic back pain is limited, complicating referral decisions [47]. Self-care referrals for these patients fails to address their functional needs, and as symptoms persist, they often seek SHC at Point B because of functional insufficiency rather than life-threatening deterioration. To improve efficiency, ambulance clinicians should shift from gatekeeping to navigating, enabling direct referral of ‘yellow line’ patients to PHCs or physiotherapists, thereby bridging the gap between emergency care and self-care.

### Limitations

First, the specific reasons why patients sought SHC could not be determined. It was not possible to distinguish whether these contacts were due to deterioration, planned health care visits, insufficient improvement, difficulties establishing a trusting relationship with ambulance clinicians, or adherence to safety-netting advice to seek further assessment if symptoms worsened.

Second, methodological limitations were present regarding the management of missing VS data. For these missing observations, we assumed a standard normal distribution, which, given the standardisation procedure, implies that missing values were more likely to represent typical rather than extreme deviations from the average patient. This assumption was based on the hypothesis that ambulance clinicians are less likely to document VS data when findings appear clinically unremarkable. However, this may not be accurate and could lead to underestimation of physiological risk, potentially influencing the predictive strength of specific variables.

Third, repeat assignments from so-called frequent callers (i.e. patients that were referred to self-care by the ambulance service more than once within 30 days) were excluded from the analysis because they did not meet the referral-frequency inclusion criteria. While this was necessary to maintain the stability of the multilevel model and avoid statistical bias from individual outliers, it limits the generalisability of the findings to patients with recurrent contact patterns. It is possible that the safety and outcomes of self-care referrals differ for this subgroup, warranting further investigation.

Forth, there is a risk of geographic bias related to variation in ADSS documentation. Although the dataset included three regions with standardised referral processes and clinical guidelines, documented ADSS use differed substantially: approximately 85% of assessments in one region, 25% in another, and no explicit documentation in the third. This uneven distribution introduces a risk of regional confounding. Although adjustments were made for known regional differences, the observed associations may partly reflect broader clinical practice variation rather than the efficacy of the ADSS itself. Nevertheless, the overall uniformity of institutional frameworks and training across regions supports the interpretation that the findings relate primarily to differences in system implementation. Even so, caution is warranted when considering generalisability.

Fifth, although 30-day mortality is a standard research endpoint in emergency care research, establishing causal links to the initial assessment is challenging, as mortality over this period may be influenced by numerous confounders.

Finally, the mortality analysis was limited by the relatively low frequency of events, which necessitates cautious interpretation of these results.

### Practice implications

Practice and policy should prioritise flexible, validated care pathways, such as direct referral to PHCs or physiotherapy, alongside enhanced decision-support tools and targeted training to improve detection of subtle deterioration and functional limitations during on-scene assessments. Nuanced clinical judgment should complement the ADSS to help reduce unnecessary ED conveyance. Strategies and improved tools should incorporate assessments of functional capacity, social support, and health literacy to ensure safe and equitable decisions and address geographic and socioeconomic disparities to prevent delayed escalation and prolonged patient distress.

## Conclusions

This study identified predictors of SHC and mortality among patients referred to self-care by ambulance clinicians, providing crucial insights to improve patient safety and care equity. Older patients demonstrated higher vulnerability, with higher risks of deterioration, mortality, and repeated SHCs. Consequently, a key conclusion is that ambulance clinicians should exercise heightened caution when considering non-conveyance for patients aged over 80 years, given the complexity, frailty, and reduced physiological reserve common in this age group. Although self-care referrals are generally safe, the high rate of re-engagement within 72 h indicates system inefficiencies and the need to review current pathways. Referral decisions extend beyond clinical stability and require legal and ethical consideration of patients’ capacity for self-care and their ability to assume, in their vulnerability, the responsibility retroceded to them. Whilst existing decision-support systems, such as the ADSS, may reduce ED conveyance, they cannot replace nuanced clinical judgment, which is inherently shaped by organisational culture and team composition. These limitations threaten care equity, as rural patients, those with limited health literacy, and older adults with frailty risk delayed escalation and prolonged distress. Future research should include patient perspectives to clarify whether SHCs result from clinical deterioration or unmet expectations to inform the ambulance service’s evolving role as a primary healthcare assessor and system navigator.

## Supplementary Information

Below is the link to the electronic supplementary material.

Supplementary Material 1: Vital signs and normative frameworks according to RETTS©. Comprehensive overview of vital signs and normative frameworks according to RETTS©. The figure details the physiological parameter thresholds used within the Rapid Emergency Triage and Treatment System (RETTS©). It includes age-specific reference ranges for paediatric cohorts ranging from newborns (0–2 months) to adolescents (12–18 years), standard values for adult patients (> 18 years), and specialized parameters for obstetric (pregnant) patients.

Supplementary Material 2: Complete aggregation process: assessments categories (ESS codes). The table presents a complete overview of the assessment categories and their associated Emergency Signs and Symptoms (ESS) codes. The aggregation process involved a systematic categorisation of ESS codes into specific clinical domains (e.g., Circulatory, Respiratory, Surgical, and Psychiatric). All authors participated actively in this process, ensuring that each ESS code was accurately mapped to its respective assessment category through a collaborative consensus-based approach.

Supplementary Material 3: Stan model code. The multilevel logistic regression model as implemented in Stan (https://mc-stan.org/).

Supplementary Material 4: Posterior predictive checks (adults). Simulated probabilities of outcomes generated from the posterior distribution for a cohort identical to that used for model fitting.

Supplementary Material 5: Posterior predictive checks (children). Simulated probabilities of outcomes generated from the posterior distribution for a cohort identical to that used for model fitting.

Supplementary Material 6: Predicted probabilities by assessment category and odds ratios of 72 h and 30-day mortality for adults. Points indicate posterior means, and horizontal lines represent 95% credibility intervals.

## Data Availability

The datasets supporting the conclusions of this article are included within the article (and its additional file(s). However, restrictions apply the availability of these data, which were used under licence for this current study, and so are not publicly available. De-identified participant data are available from the authors upon reasonable request and with permission from the participating ambulance service organisations.

## Footnotes

1 The patient care pathway was not followed in the study, which meant that a patient could have used several levels of care contacts and be included in several of the explored outcomes.

## Abbreviations

ACVPU: Alert, Confusion, Verbal, Pain or Unresponsive
ADSS: Advisory decision support system
CI: Credible intervals
DeSO: Demographic statistics area
ED: Emergency department
EMCC: Emergency medical communication centre
ESS: Early signs and symptoms
IQR: Interquartile range
OR: Odds ratio
PHC: Primary healthcare centre
PP: Predicted probability
RETTS: Rapid Emergency Triage and Treatment System
RLS: Reaction Level Scale
ROBUST: Reporting Of Bayes Used in Clinical Studies
SHC: Subsequent healthcare contact
STROBE: Strengthening the Reporting of Observational Studies in Epidemiology
VS: Vital signs
