# Supplementary figures and images for: Patient safety and predictors for subsequent healthcare contact after self-care referral from Swedish ambulance services: a retrospective cohort study
Source: BMC Emerg Med. 2026 Apr 2;26:100. doi: 10.1186/s12873-026-01561-4 (PMC13063699; doi:10.1186/s12873-026-01561-4)

Additional file 1. Vital signs and normative frameworks according to RETTS©


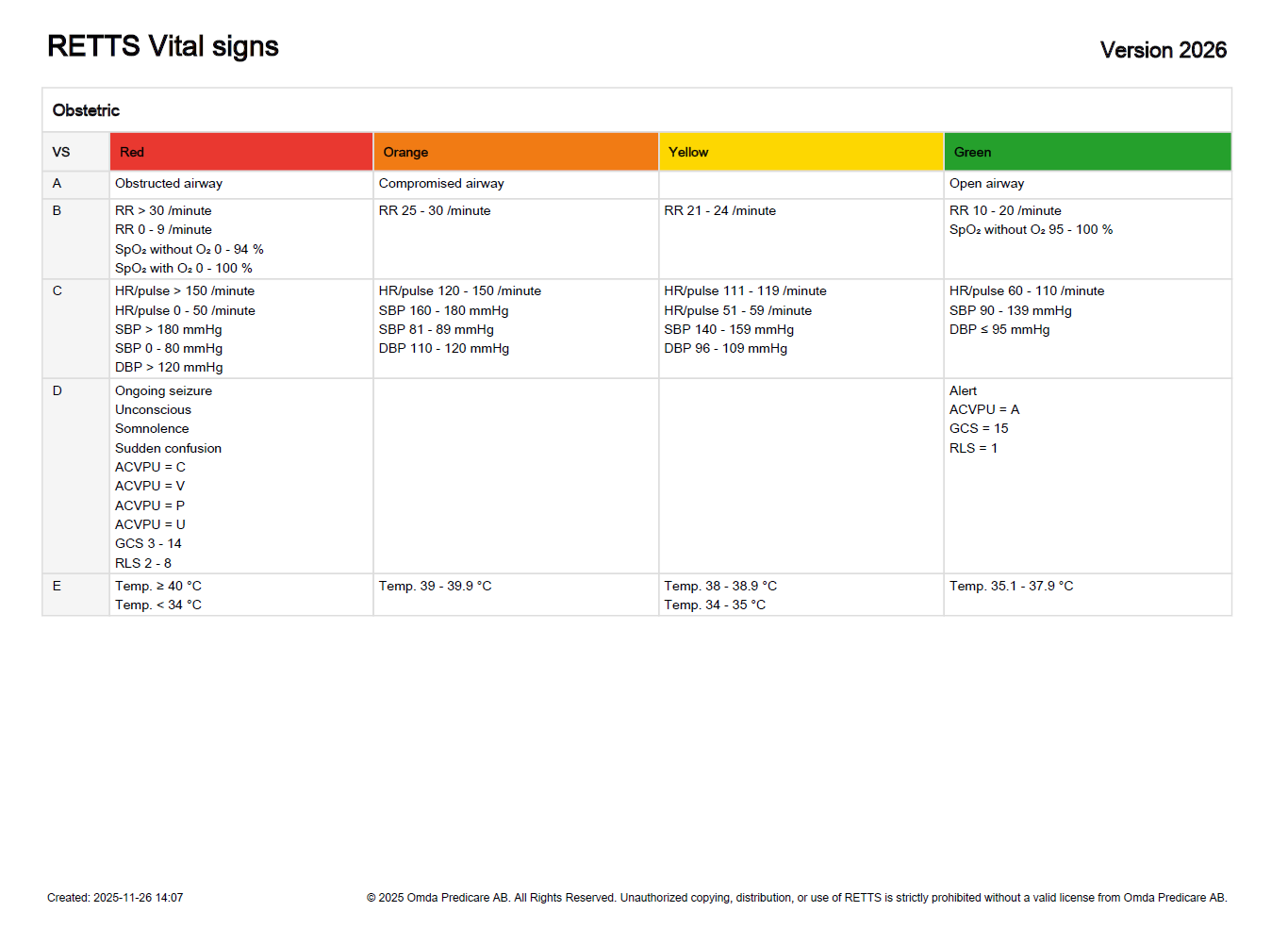


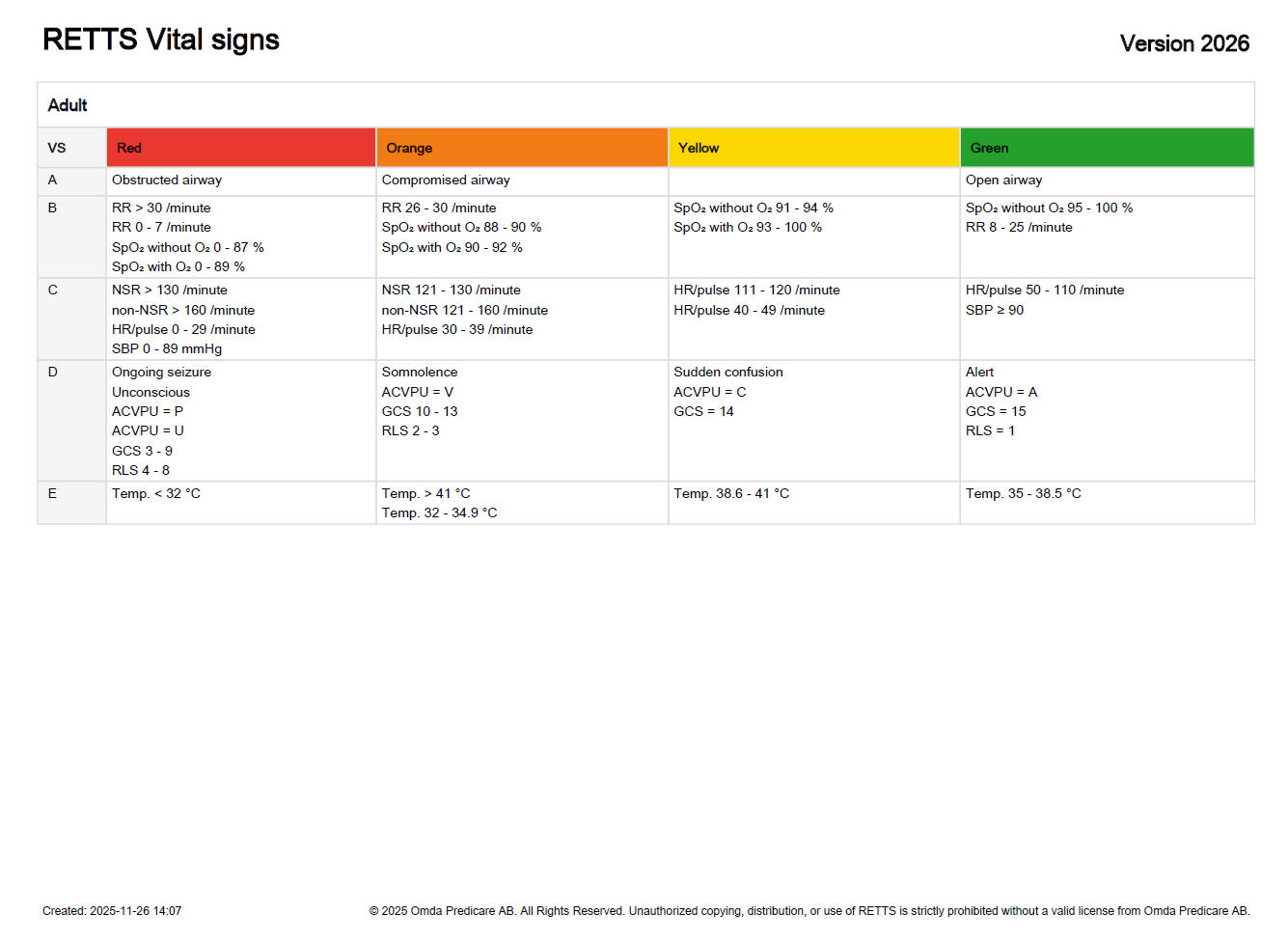


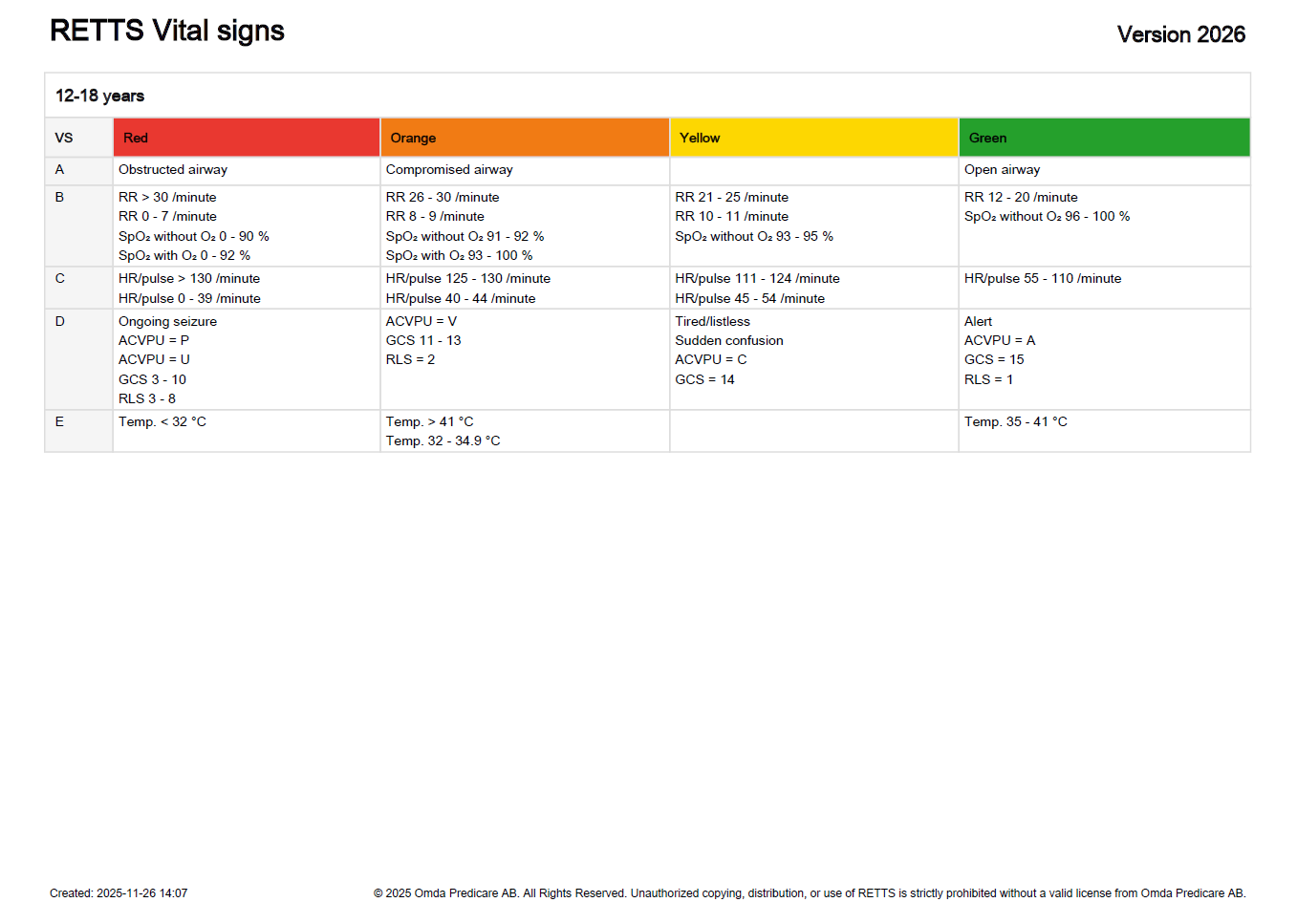


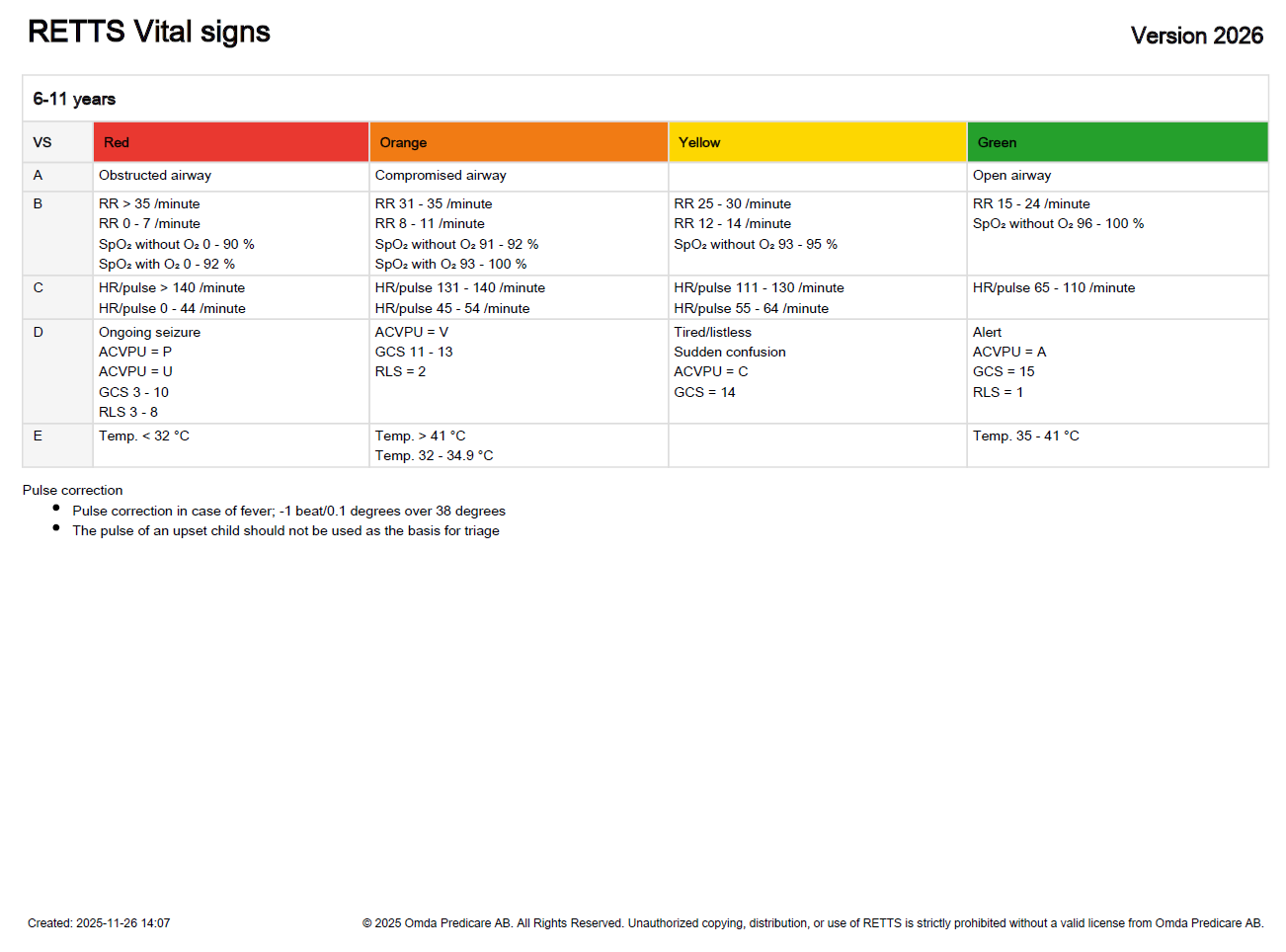


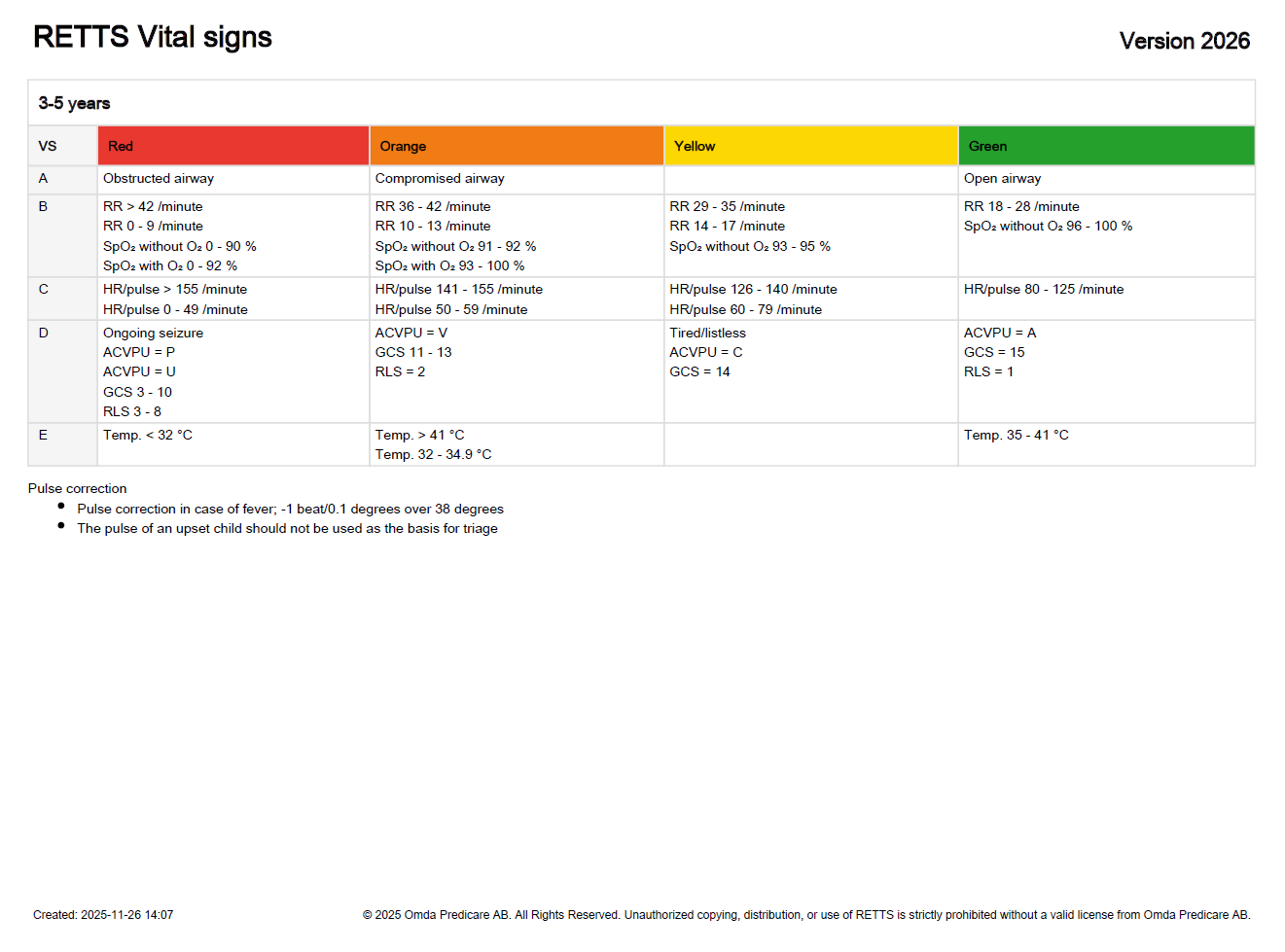


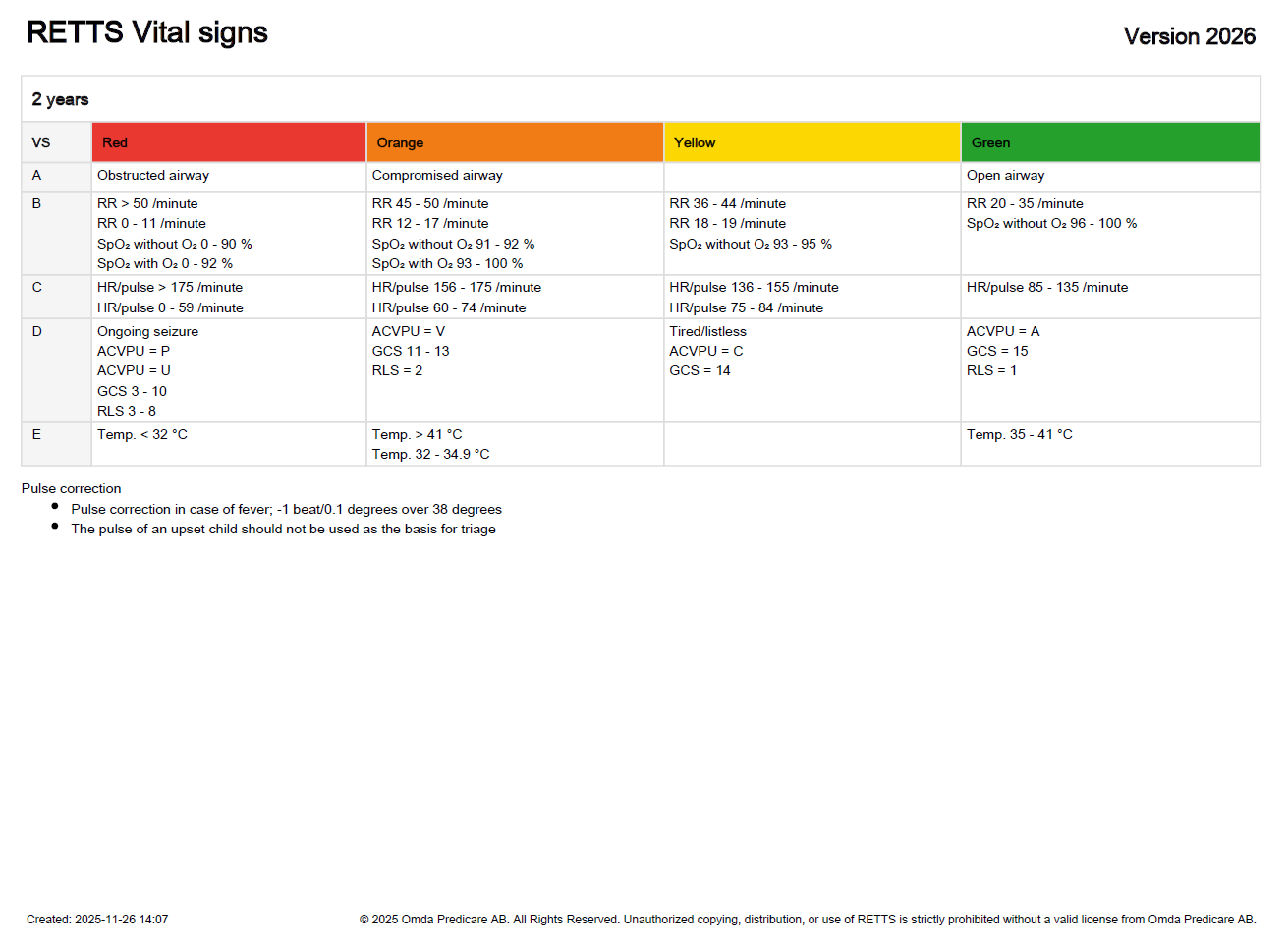


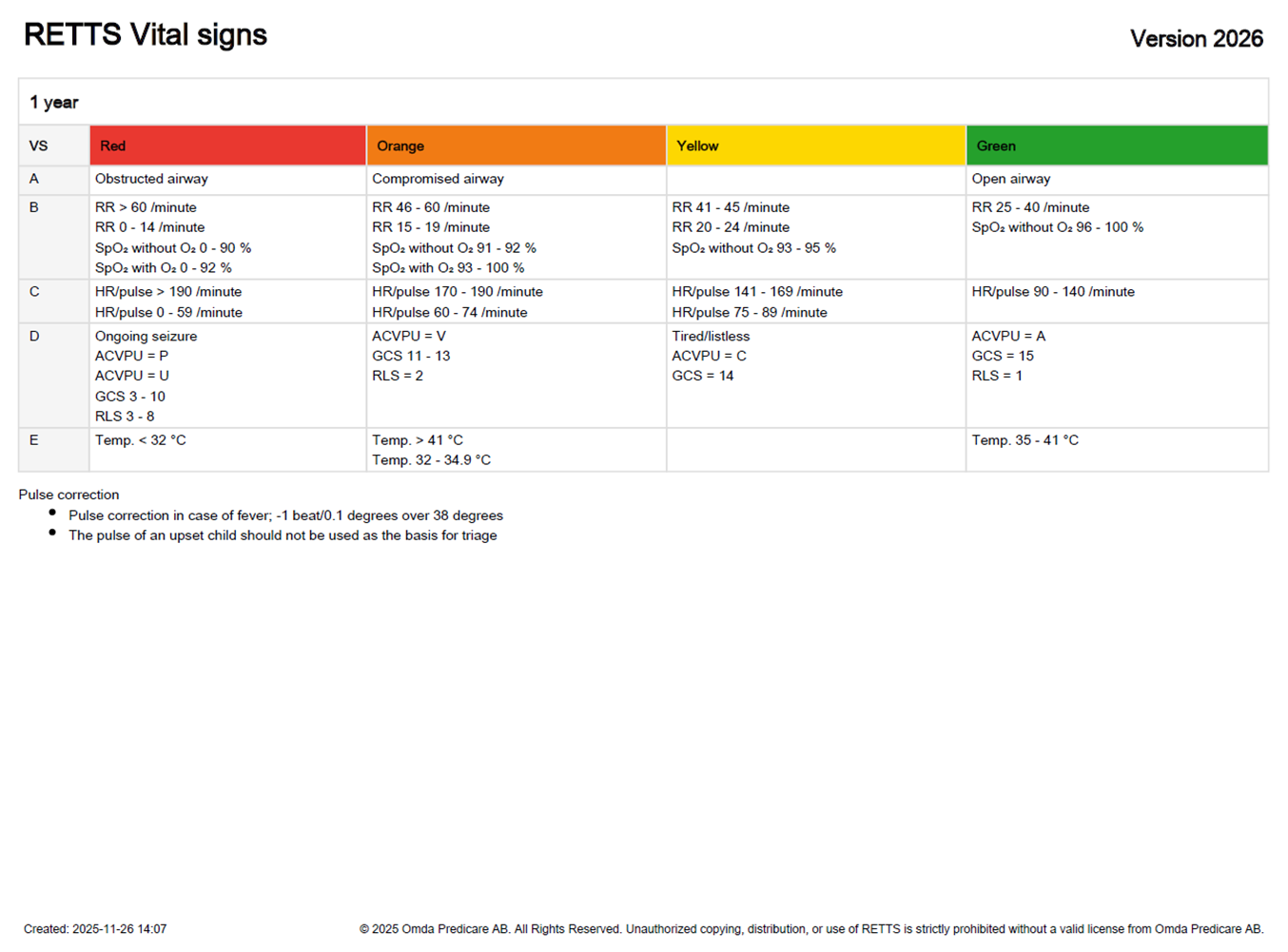


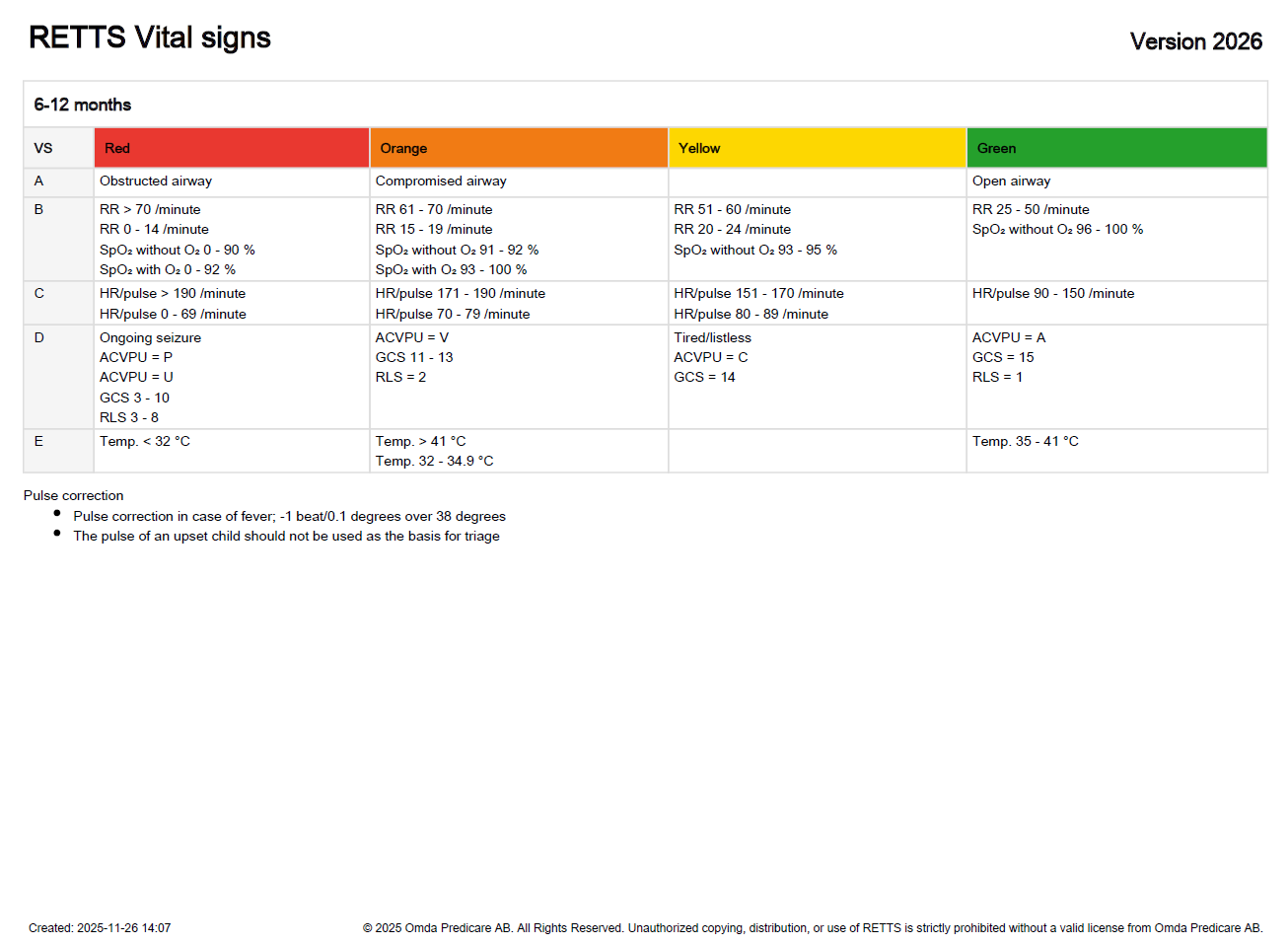


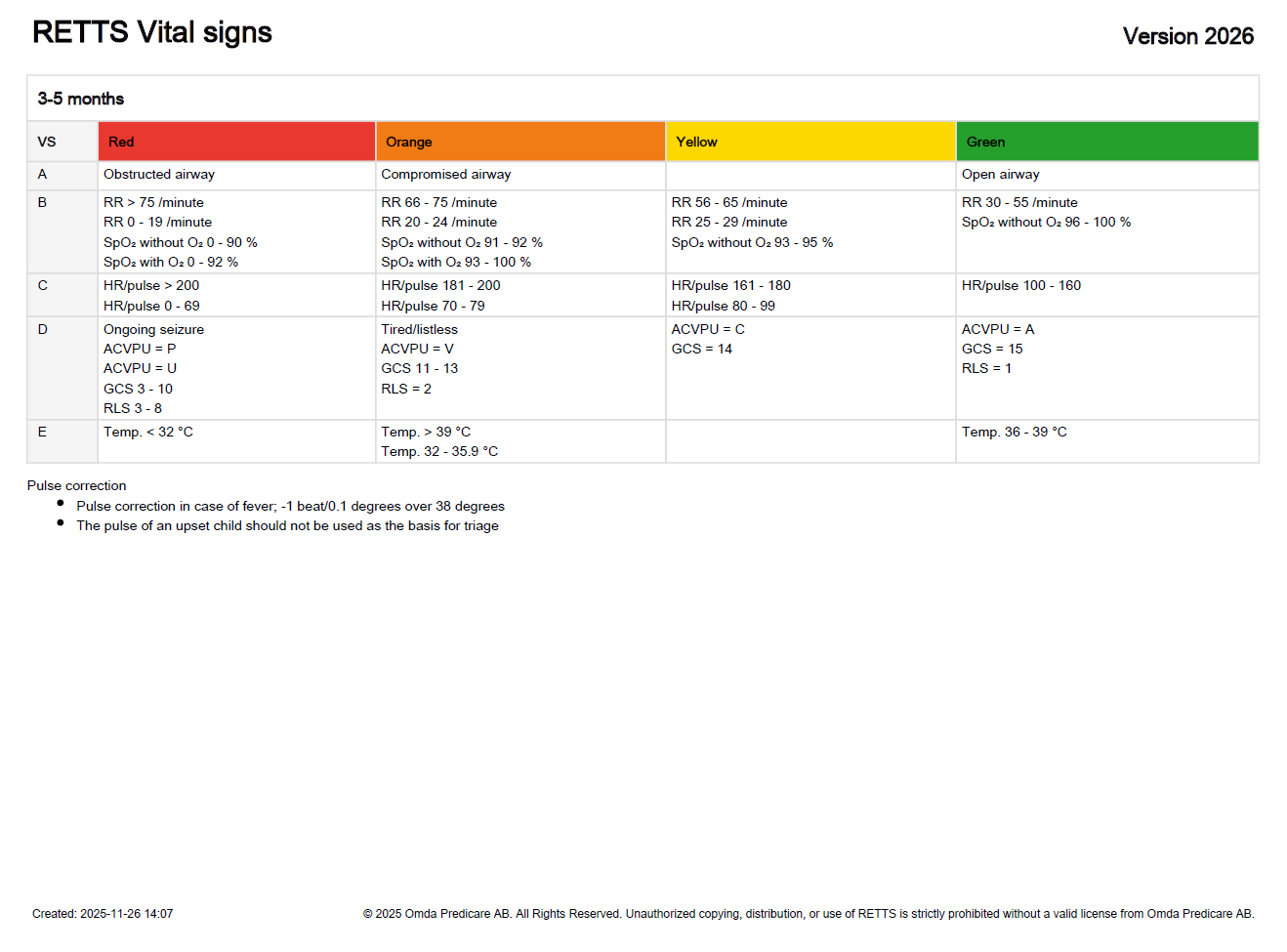


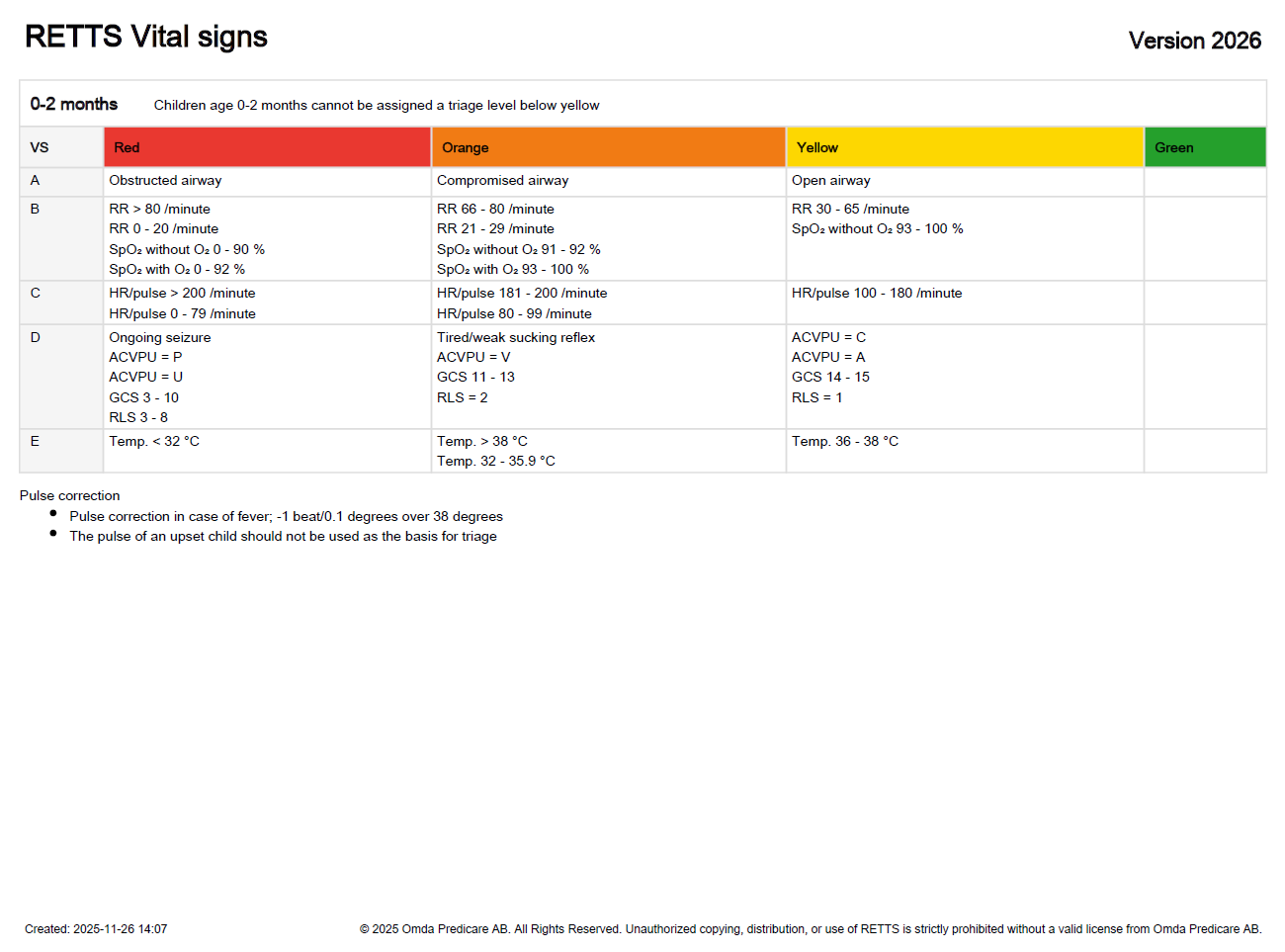

Supplement: Supplementary file 1 — Supplementary Material 1: Vital signs and normative frameworks according to RETTS©. Comprehensive overview of vital signs and normative frameworks according to RETTS©. The figure details the physiological parameter thresholds used within the Rapid Emergency Triage and Treatment System (RETTS©). It includes age-specific reference ranges for paediatric cohorts ranging from newborns (0–2 months) to adolescents (12–18 years), standard values for adult patients (> 18 years), and specialized parameters for obstetric (pregnant) patients. [file 12873_2026_1561_MOESM1_ESM.docx]
