# Supplementary material for: Patient safety and predictors for subsequent healthcare contact after self-care referral from Swedish ambulance services: a retrospective cohort study
Source: BMC Emerg Med. 2026 Apr 2;26:100. doi: 10.1186/s12873-026-01561-4 (PMC13063699; doi:10.1186/s12873-026-01561-4)
Supplement: Supplementary file 2 — Supplementary Material 2: Complete aggregation process: assessments categories (ESS codes). The table presents a complete overview of the assessment categories and their associated Emergency Signs and Symptoms (ESS) codes. The aggregation process involved a systematic categorisation of ESS codes into specific clinical domains (e.g., Circulatory, Respiratory, Surgical, and Psychiatric). All authors participated actively in this process, ensuring that each ESS code was accurately mapped to its respective assessment category through a collaborative consensus-based approach. [file 12873_2026_1561_MOESM2_ESM.docx]

| ESS Code | Symptom / Description | Assessment category | Super category |
| --- | --- | --- | --- |
| 1 | Atrial fibrillation, abnormal heart rhythm | Circulatory | Adult |
| 3 | Haemoptysis, epistaxis, post-operative tonsillar bleeding | Surgical | Adult |
| 4 | Dyspnoea/shortness of breath, hyperventilation | Respiratory | Adult |
| 5 | Chest pain, pleuritic chest pain | Circulatory | Adult |
| 6 | Abdominal/flank/stomach pain, gastrointestinal issues, swelling | Surgical | Adult |
| 7 | GI bleeding (melena/hematemesis), rectal issues, haemorrhoids | Surgical | Adult |
| 8 | Nausea, vomiting, diarrhoea | Surgical | Adult |
| 9 | Seizures, known epilepsy | Neurology | Adult |
| 11 | Dizziness, balance problems | Neurology | Adult |
| 12 | Facial paralysis, neurological deficit, TIA, suspected stroke | Neurology | Adult |
| 13 | Joint pain without trauma / non-traumatic arthralgia | Orthopaedic | Adult |
| 14 | Non-traumatic back, chest, or neck pain | Orthopaedic | Adult |
| 15 | Extremity issues, pain, swelling, lower leg oedema | Circulatory | Adult |
| 16 | Haematuria, dysuria, urinary retention, urinary problems | Gynaecology / Urology | Adult |
| 17 | Scrotal pain/swelling, penile/urethral issues | Gynaecology / Urology | Adult |
| 19 | Headache, neuralgia/nerve pain | Neurology | Adult |
| 20 | Loss of consciousness, syncope/fainting | Neurology | Adult |
| 21 | Pregnancy, gynaecological issues, pelvic pain, vaginal bleeding | Gynaecology / Urology | Adult |
| 29 | Ascites, jaundice/icterus | Surgical | Adult |
| 30 | Hanging/strangulation, jaw/face/nose/neck/head injury | Trauma | Adult |
| 31 | Abdominal, genital, or thoracic/back injury | Trauma | Adult |
| 32 | Hematoma, swelling, bruise, abrasion, laceration, wound injury | Trauma | Adult |
| 33 | Shoulder/clavicle injury, arm/wrist injury, hand/finger injury | Trauma | Adult |
| 34 | Foot/toe injury, hip/femur/pelvis injury, knee/lower leg injury | Trauma | Adult |
| 35 | Lightning/burn/electrical/chemical/cold/radiation/CO injury | Trauma | Adult |
| 36 | Drowning/near-drowning, suspected decompression sickness | Trauma | Adult |
| 37 | Eye injury | Trauma | Adult |
| 38 | General trauma | Trauma | Adult |
| 40 | Poisoning / intoxication | Medical | Adult |
| 41 | Animal/human bites and stings, toxic effects of animals | Medical | Adult |
| 42 | Physical abuse, sexual assault | Trauma | Adult |
| 43 | Allergy, rash, itching, medical reaction | Medical | Adult |
| 44 | Mouth blisters, sore throat, common cold, cough | Medical | Adult |
| 45 | Ear problems, earache/otalgia | Eye, ear, nose, throat | Adult |
| 46 | Foreign body in oesophagus, airway, nose, or ear | Eye, ear, nose, throat | Adult |
| 47 | Fever, infection, local skin infection, suspected sepsis | Infection | Adult |
| 49 | Known diabetes, hyperglycaemia/high blood sugar | Medical | Adult |
| 50 | Hypoglycaemia / low blood sugar | Medical | Adult |
| 51 | Addison's disease, known adrenal insufficiency | Medical | Adult |
| 53 | Tendency to fall, hypertension, malaise/fatigue, PEG issues | Medical | Adult |
| 57 | Eye infection, eye problems | Infection | Adult |
| 65 | Cardiac arrest | Circulatory | Adult |
| 71 | Gypsum (cast) problem | Orthopaedic | Adult |
| 72 | Postoperative complication | Surgical | Adult |
| 80 | Depressive episode | Psychiatric | Adult |
| 81 | Hypomania, mania | Psychiatric | Adult |
| 82 | Panic disorder, anxiety | Psychiatric | Adult |
| 83 | Sleep disorder | Psychiatric | Adult |
| 84 | Psychotic symptoms | Psychiatric | Adult |
| 85 | Substance abuse/dependence, tremors | Psychiatric | Adult |
| 86 | Self-harm | Psychiatric | Adult |
| 87 | Eating disorder | Psychiatric | Adult |
| 88 | Crisis reaction, reaction to severe stress | Psychiatric | Adult |
| 89 | Dementia, disoriented, organic mental disorders | Psychiatric | Adult |
| 90 | Non-acute psychiatric issue | Psychiatric | Adult |
| 91 | Organic catatonia | Psychiatric | Adult |
| 98 | Behaviour disorder, risk assessment | Psychiatric | Adult |
| 99 | Suicide risk | Psychiatric | Adult |

| ESS code | Symptom / Description | Assessment category | Super  category |
| --- | --- | --- | --- |
| 101 | Abnormal heart rhythm | Circulatory | Child |
| 103 | Haemoptysis, epistaxis, post-operative tonsillar bleeding | Surgical | Child |
| 104 | Dyspnoea/shortness of breath, apnoea, hyperventilation | Respiratory | Child |
| 105 | Chest pain | Circulatory | Child |
| 106 | Abdominal/flank/stomach pain, gastrointestinal issues, swelling | Surgical | Child |
| 107 | Melena (bloody stool), diarrhoea/loose stools, anal/rectal symptoms | Surgical | Child |
| 108 | Hematemesis (bloody vomiting), nausea, vomiting | Surgical | Child |
| 109 | Known epilepsy, absence seizure, seizures/convulsions, infantile spasms | Neurology | Child |
| 111 | Dizziness/balance problems | Neurology | Child |
| 112 | Facial paralysis, neurological deficit | Neurology | Child |
| 113 | Limp without trauma, joint pain without trauma/non-traumatic arthralgia | Orthopaedic | Child |
| 114 | Back pain without trauma/non-traumatic back pain | Orthopaedic | Child |
| 115 | Extremity pain, extremity swelling, oedema | circulatory | Child |
| 116 | Haematuria (blood in urine), dysuria, urinary retention, urinary problems | Surgical | Child |
| 117 | Scrotal pain, scrotal swelling, penile/urethral issues | Gynaecology, urology | Child |
| 119 | Headache | Neurology | Child |
| 120 | Loss of consciousness, syncope/fainting | Neurology | Child |
| 121 | Pregnancy, gynaecological issues, pelvic issues, vaginal bleeding | Gynaecology, urology | Child |
| 129 | Jaundice/icterus | Surgical | Child |
| 130 | Hanging/strangulation, jaw injury, head/face/nose injury | Trauma | Child |
| 131 | Genital injury, thoracic/back injury | Trauma | Child |
| 133 | Shoulder/clavicle injury, arm/wrist injury, hand/finger injury | Trauma | Child |
| 134 | Foot/toe injury, hip/femur/pelvis injury, knee/lower leg injury | Trauma | Child |
| 135 | Lightning/burn/electrical/chemical/cold/radiation/CO injury | Trauma | Child |
| 136 | Drowning/near-drowning, suspected decompression sickness | Trauma | Child |
| 137 | Eye injury | Eye, ear, nose, throat | Child |
| 138 | General trauma | Trauma | Child |
| 140 | Poisoning/intoxication | Medical | Child |
| 141 | Human/animal bites and stings, puncture wound with infection risk | Medical | Child |
| 142 | Suspected physical abuse, suspected sexual assault | Trauma | Child |
| 143 | Allergy, exanthema/skin rash/bruise, itching/pruritus, medical reaction | Medical | Child |
| 144 | Mouth blisters, sore throat/common cold, cough | Medical | Child |
| 145 | Ear problems, earache/otalgia | Eye, ear, nose, throat | Child |
| 146 | Foreign body in oesophagus, airway, nose, or ear | Eye, ear, nose, throat | Child |
| 147 | Local skin infection incl. abscess/lymphadenitis, dental infection | Infection | Child |
| 148 | Anaemia, blood disorders | Medical | Child |
| 149 | Hyperglycaemia/high blood sugar | Medical | Child |
| 150 | Hypoglycaemia/low blood sugar | Medical | Child |
| 151 | Addison's disease, known adrenal insufficiency | Medical | Child |
| 153 | Crying child, worried parents, poor weight gain/fluid intake/nutrition | Medical | Child |
| 154 | Fever of unknown origin/Fever without source | Infection | Child |
| 155 | Known diabetes, metabolic disorders | Medical | Child |
| 157 | Eye infection, eye problems | Infection | Child |
| 165 | Cardiac arrest | Infection | Child |
| 171 | Gypsum (cast) problem | Orthopaedic | Child |
| 172 | Postoperative complication | Surgical | Child |
| 180 | Depressive episode, depressed mood | Psychiatric | Child |
| 181 | Hypomania, mania | Psychiatric | Child |
| 182 | Anxiety | Psychiatric | Child |
| 183 | Sleep disorder | Psychiatric | Child |
| 184 | Psychotic symptoms | Psychiatric | Child |
| 185 | Substance abuse/dependence | Psychiatric | Child |
| 187 | Eating disorder | Psychiatric | Child |
| 188 | Crisis reaction, reaction to severe stress | Psychiatric | Child |
| 189 | Mental ill-health/Mental health issues | Psychiatric | Child |
| 190 | Non-acute psychiatric issue | Psychiatric | Child |
| 191 | Organic catatonia | Psychiatric | Child |
| 198 | Behaviour disorder, risk assessment | Psychiatric | Child |
| 199 | Self-harm, suicide risk assessment | Psychiatric | Child |

| ESS  Code | Symptom / Description | Assessment category | Super  category |
| --- | --- | --- | --- |
| 204 | Dyspnoea/shortness of breath in pregnancy, hyperventilation | Respiratory | Pregnant |
| 205 | Chest pain in pregnancy, pleuritic chest pain | Circulatory | Pregnant |
| 206 | Abdominal/flank pain, pregnancy-related abdominal pain | Surgical | Pregnant |
| 207 | Hyperemesis, nausea/vomiting in pregnancy, diarrhoea | Surgical | Pregnant |
| 209 | Known epilepsy in pregnancy, seizures in pregnancy | Neurology | Pregnant |
| 211 | Dizziness/balance problems in pregnancy | Neurology | Pregnant |
| 212 | Facial paralysis/stroke/neurological deficit in pregnancy | Neurology | Pregnant |
| 215 | Extremity issues, pain, swelling, lower leg oedema in pregnancy | Circulatory | Pregnant |
| 219 | Headache in pregnancy | Neurology | Pregnant |
| 220 | Loss of consciousness in pregnancy, syncope/fainting | Neurology | Pregnant |
| 221 | Vaginal bleeding in pregnancy | Gynaecology / urology | Pregnant |
| 222 | Postpartum bleeding (within 6 weeks) | Gynaecology / urology | Pregnant |
| 224 | Abnormal foetal heart sounds, foetal distress/decreased movements | Gynaecology / urology | Pregnant |
| 226 | Suspected rupture of membrane, active labour, contractions | Gynaecology / urology | Pregnant |
| 231 | Abdominal injury in pregnancy, thoracic/back injury | Trauma | Pregnant |
| 247 | Breastfeeding issues, breast pain, fever/infection (within 6 weeks) | Infection | Pregnant |
| 253 | Hypertension/high blood pressure, suspected preeclampsia | Medical | Pregnant |
