# Supplementary material for: Patient safety and predictors for subsequent healthcare contact after self-care referral from Swedish ambulance services: a retrospective cohort study
Source: BMC Emerg Med. 2026 Apr 2;26:100. doi: 10.1186/s12873-026-01561-4 (PMC13063699; doi:10.1186/s12873-026-01561-4)
Supplement: Supplementary file 4 — Supplementary Material 4: Posterior predictive checks (adults). Simulated probabilities of outcomes generated from the posterior distribution for a cohort identical to that used for model fitting. [file 12873_2026_1561_MOESM4_ESM.docx]

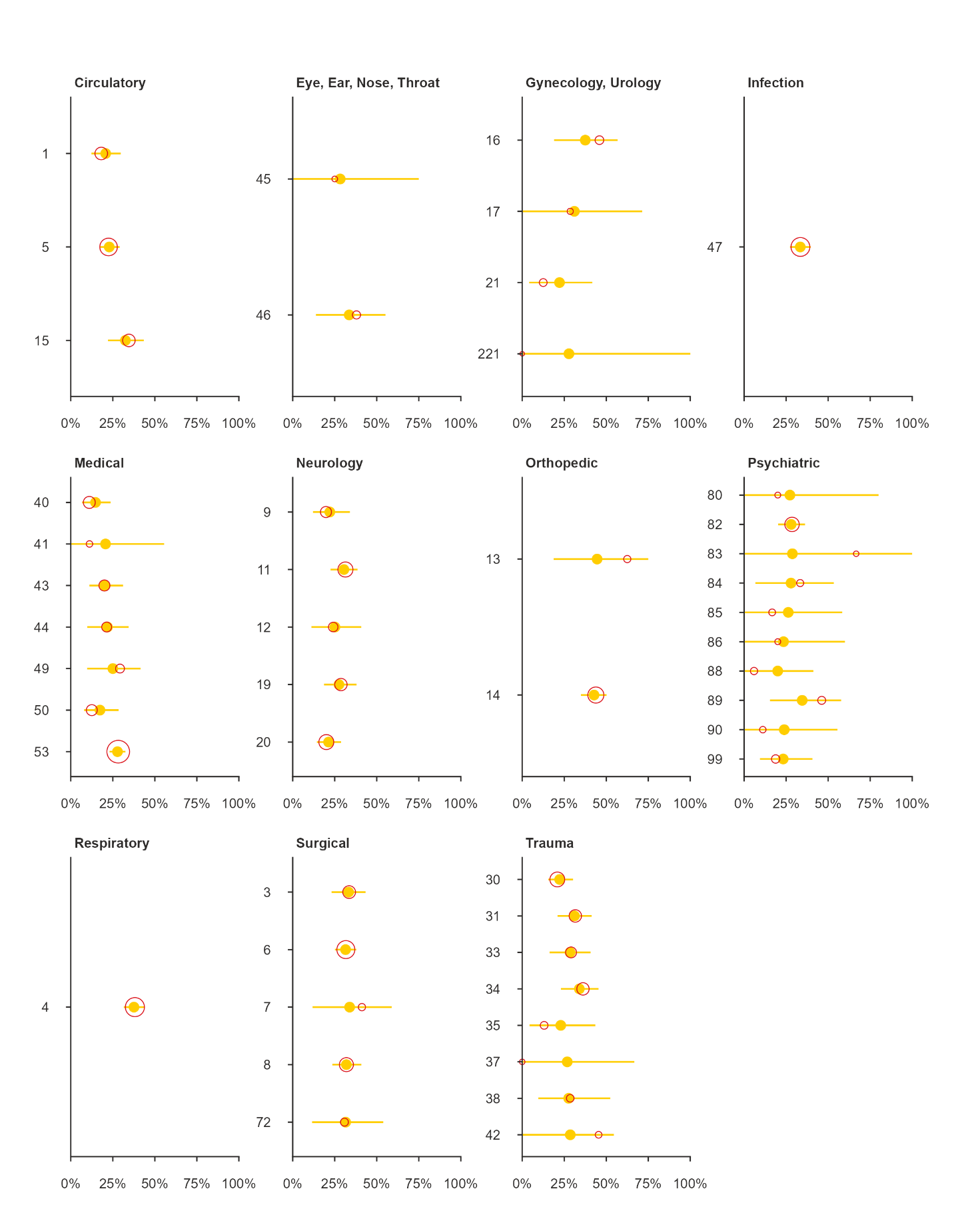


**Additional file 4. Supplementary figure 6.** Simulated probabilities of any subsequent healthcare contact within 72 h generated from the posterior distribution for a cohort identical to that used for model fitting. The points indicate posterior means, and the horizontal lines represent 95%-credibility intervals. The red circles indicate observed data and are approximately proportional to the number of observations.


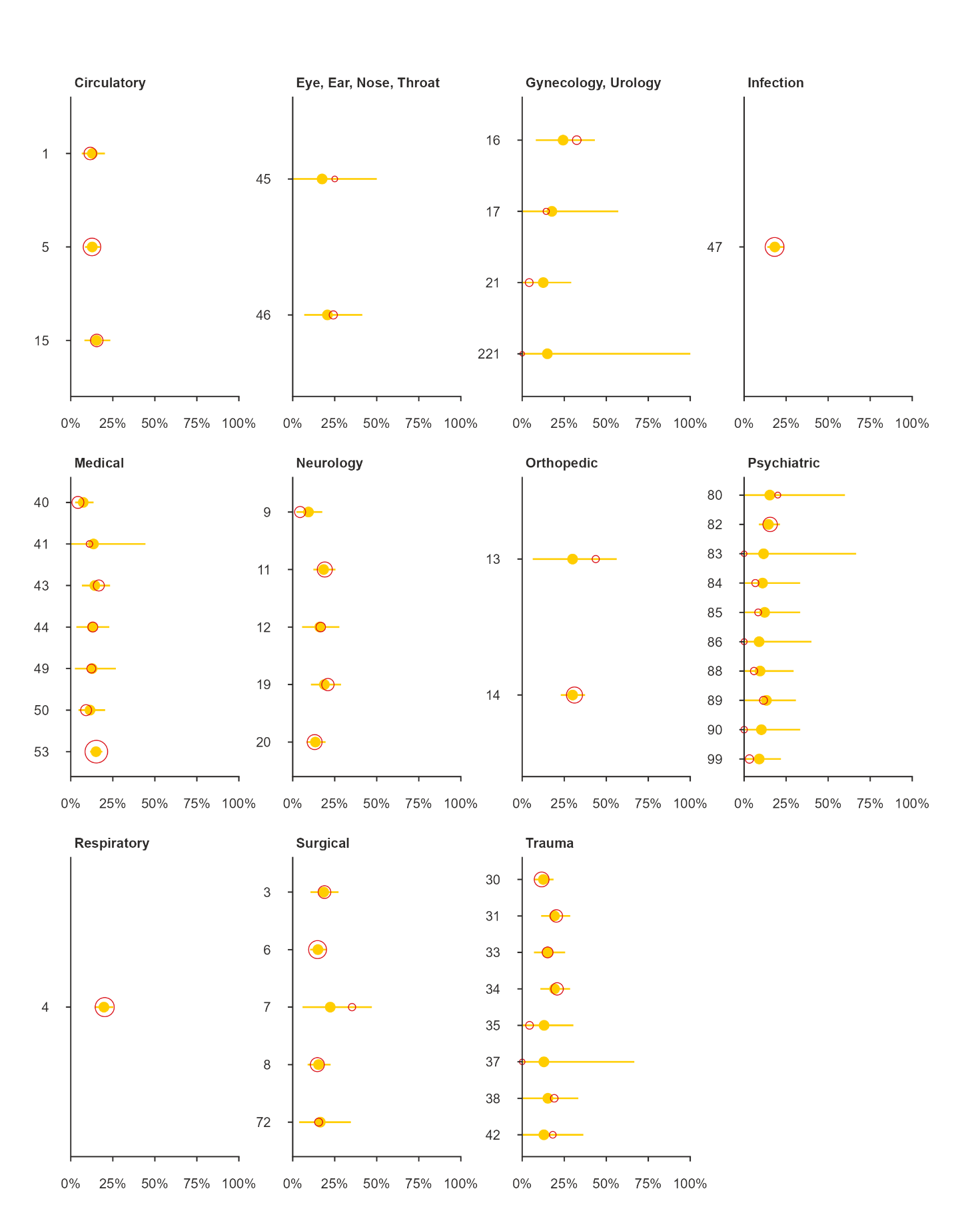


**Supplementary figure 7.** Simulated probabilities of a primary healthcare centre visit within 72 h generated from the posterior distribution for a cohort identical to that used for model fitting. The points indicate posterior means, and the horizontal lines represent 95%- credibility intervals. The red circles indicate observed data and are approximately proportional to the number of observations.


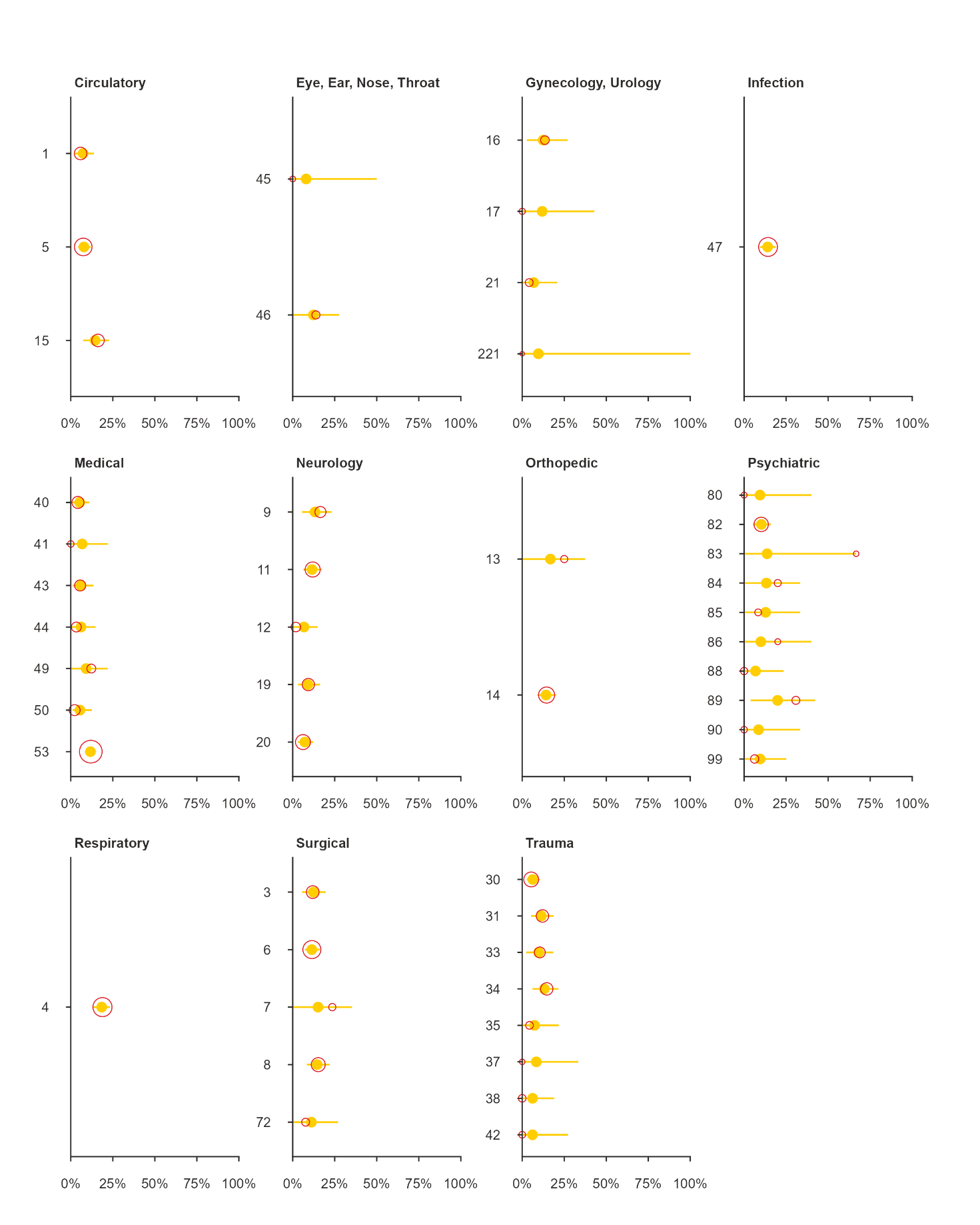


**Supplementary figure 8.** Simulated probabilities of ambulance recontact within 72 h generated from the posterior distribution for a cohort identical to that used for model fitting. The points indicate posterior means, and the horizontal lines represent 95%- credibility intervals. The red circles indicate observed data and are approximately proportional to the number of observations.


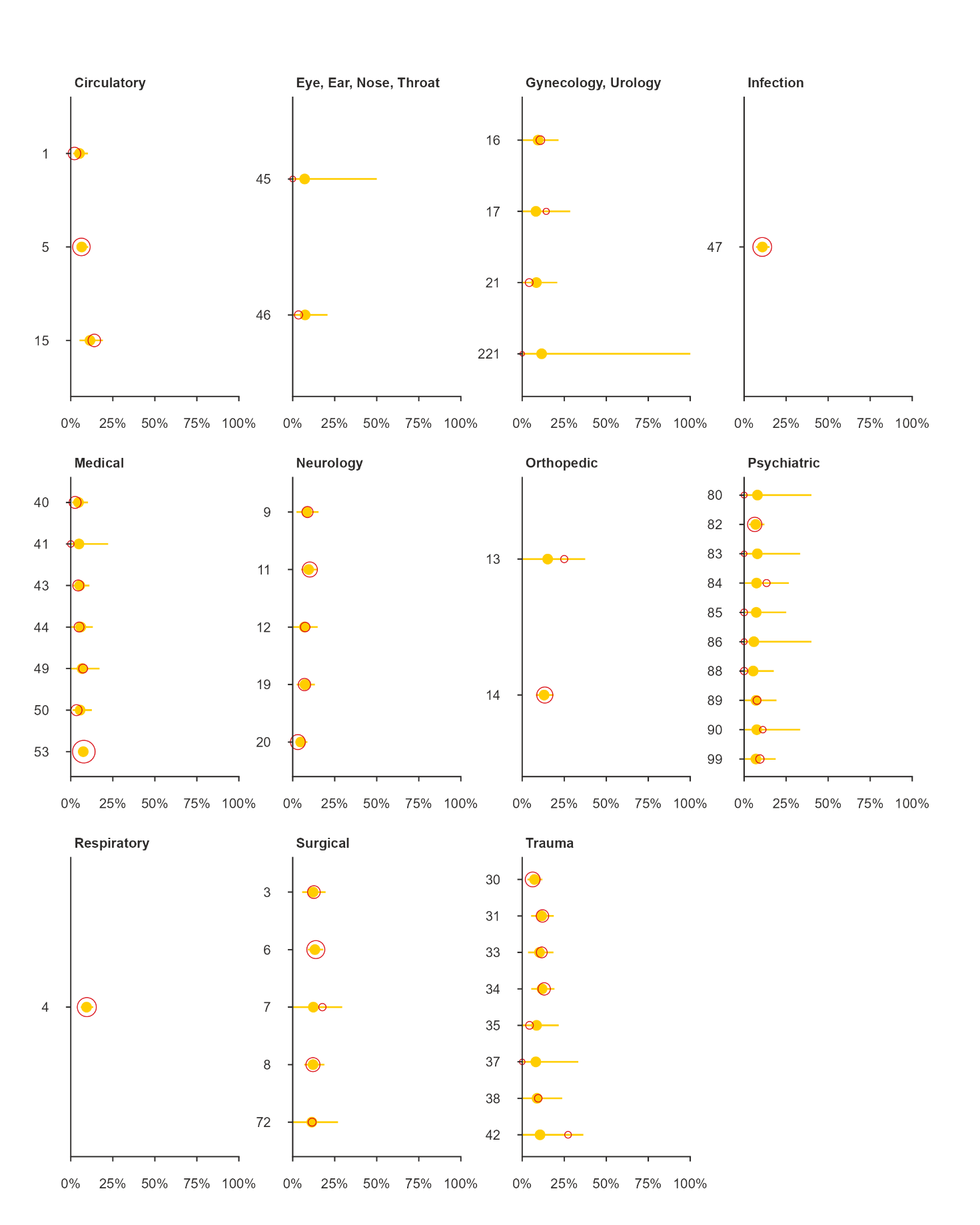


**Supplementary figure 9.** Simulated probabilities of an emergency department visit within 72 h generated from the posterior distribution for a cohort identical to that used for model fitting. The points indicate posterior means, and the horizontal lines represent 95%- credibility intervals. The red circles indicate observed data and are approximately proportional to the number of observations.


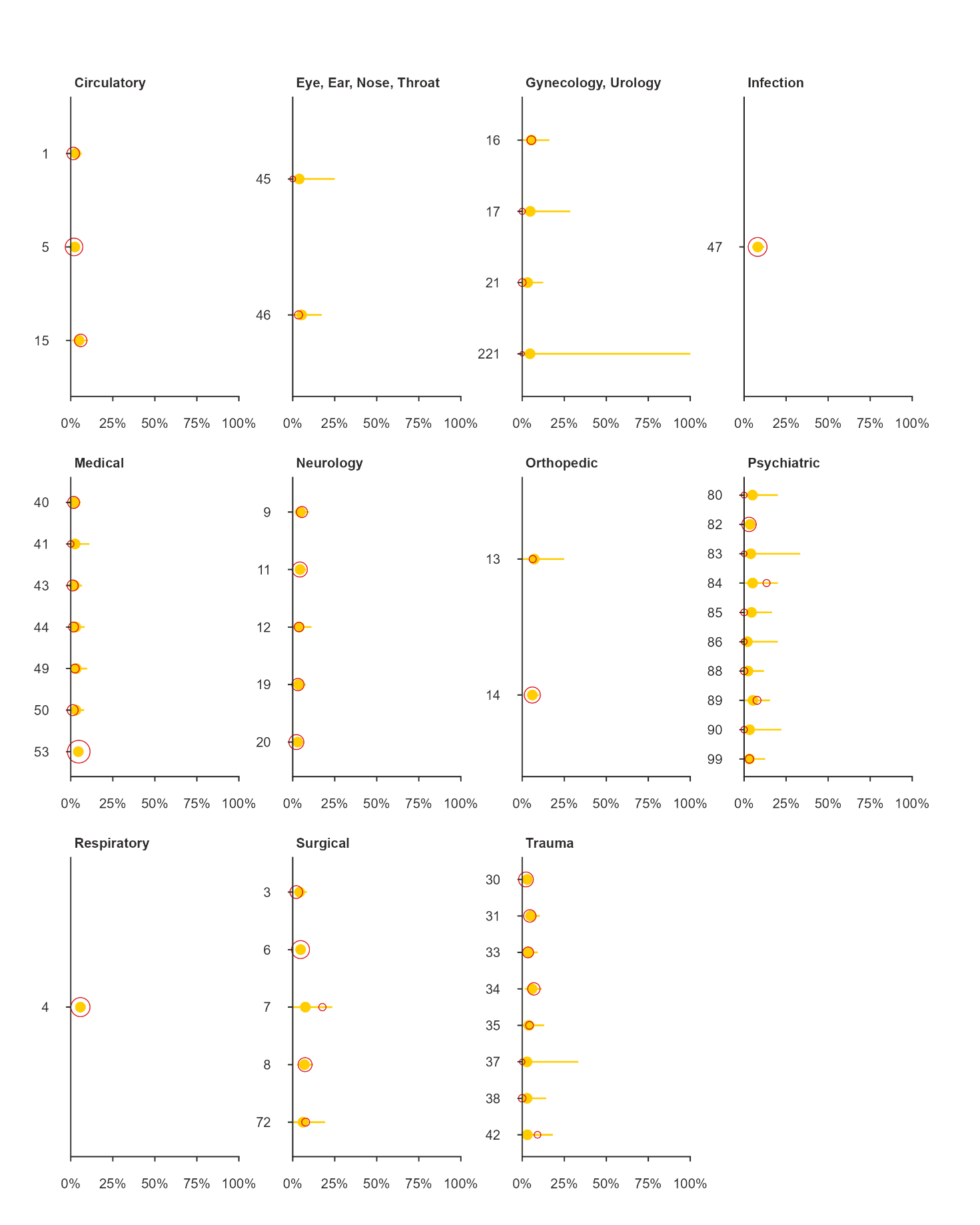


**Supplementary figure 10.** Simulated probabilities of hospitalisation within 72 h generated from the posterior distribution for a cohort identical to that used for model fitting. The points indicate posterior means, and the horizontal lines represent 95%- credibility intervals. The red circles indicate observed data and are approximately proportional to the number of observations.
