# Supplementary material for: Patient safety and predictors for subsequent healthcare contact after self-care referral from Swedish ambulance services: a retrospective cohort study
Source: BMC Emerg Med. 2026 Apr 2;26:100. doi: 10.1186/s12873-026-01561-4 (PMC13063699; doi:10.1186/s12873-026-01561-4)
Supplement: Supplementary file 6 — Supplementary Material 6: Predicted probabilities by assessment category and odds ratios of 72 h and 30-day mortality for adults. Points indicate posterior means, and horizontal lines represent 95% credibility intervals. [file 12873_2026_1561_MOESM6_ESM.docx]

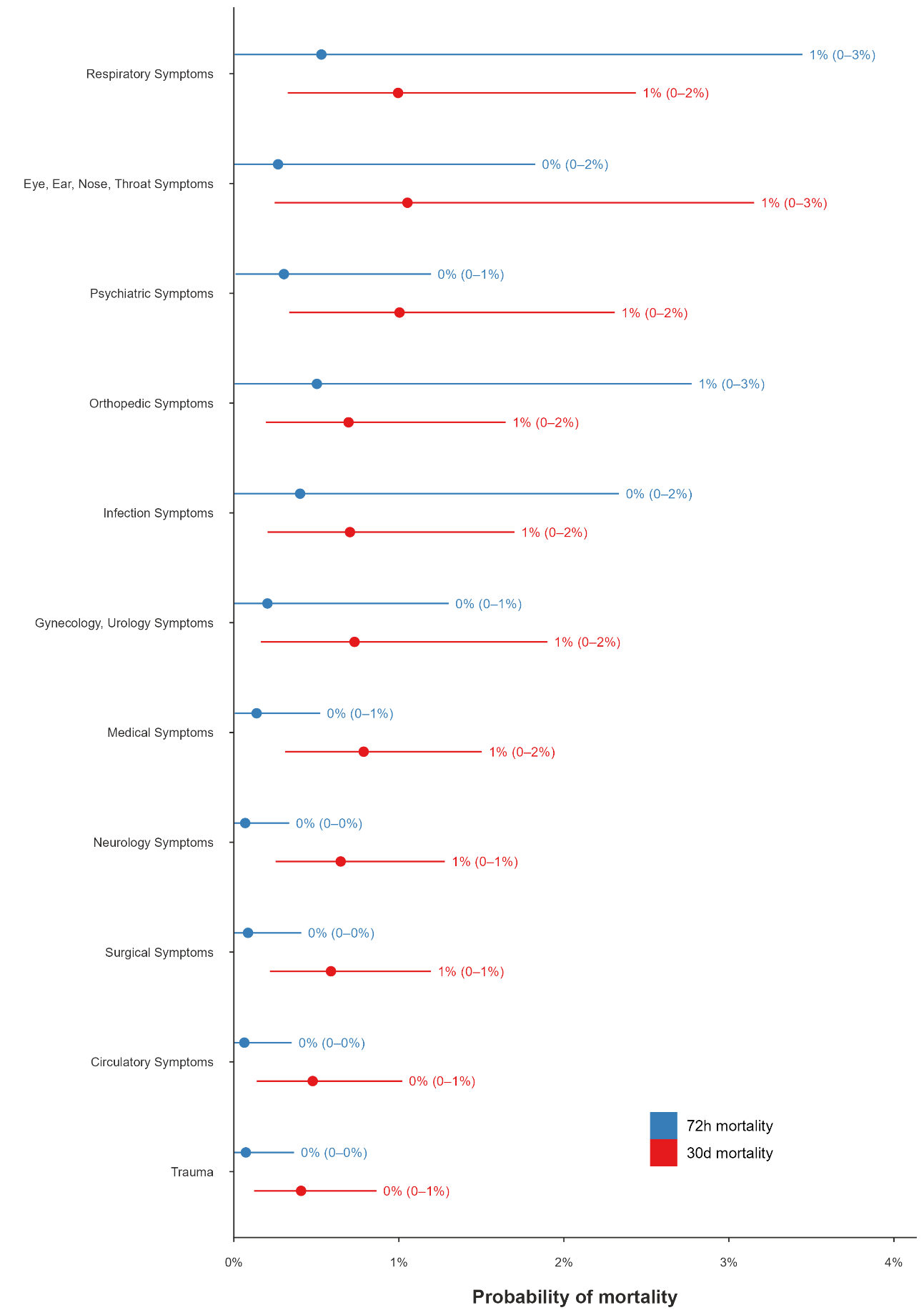


**Additional file 6. Supplementary figure 16.** Predicted probabilities by assessment category of 72 h and 30-day mortality for adults. Points indicate posterior means, and horizontal lines represent 95% credibility intervals.


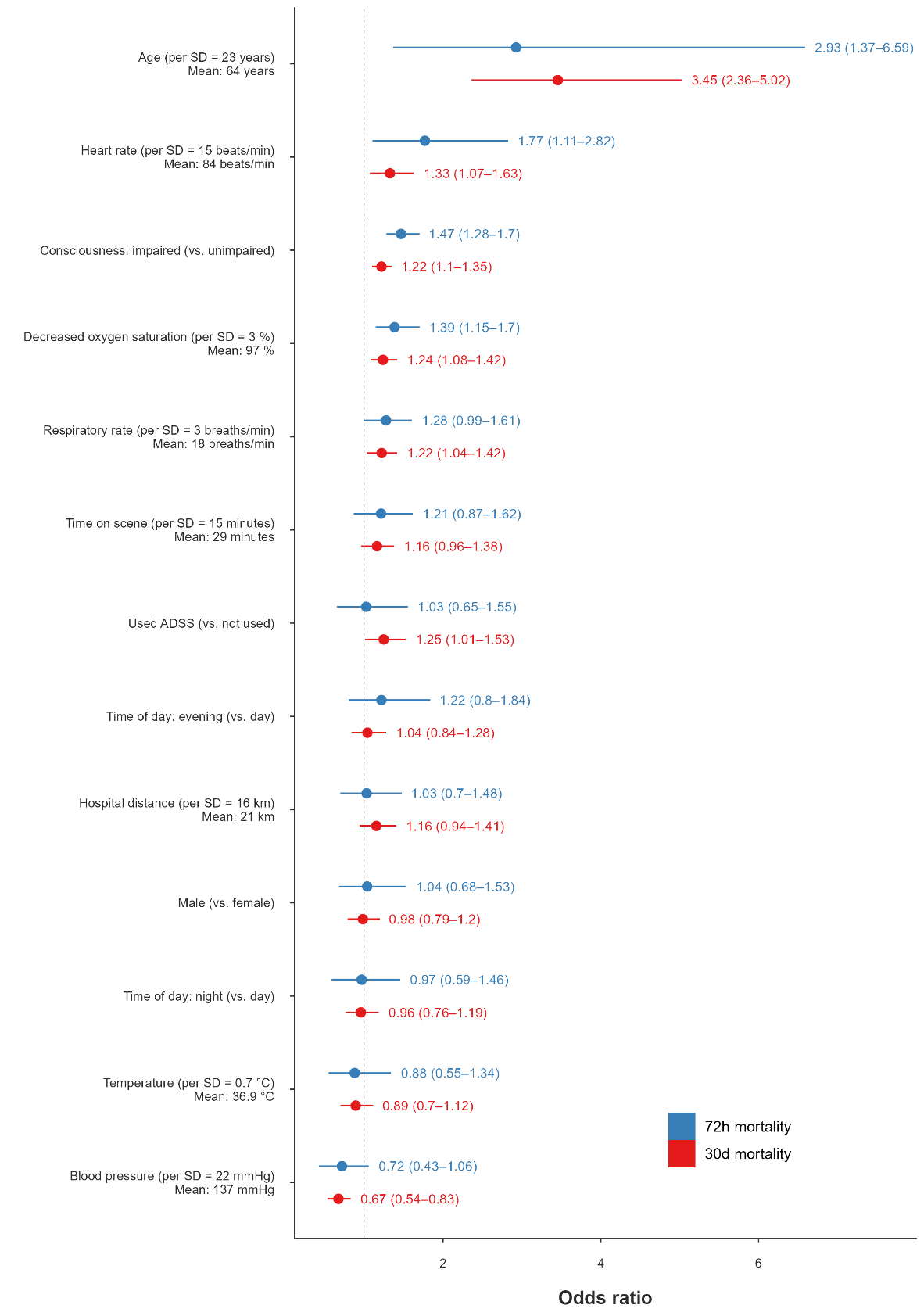


**Supplementary figure 17.** Posterior odds ratios of 72 h and 30-day mortality for adults. Points indicate posterior means, and horizontal lines represent 95%-credibility intervals.
